# Understanding dietary and staple food transitions in China from multiple scales

**DOI:** 10.1371/journal.pone.0195775

**Published:** 2018-04-24

**Authors:** Xiao Chang, Ruth S. DeFries, Liming Liu, Kyle Davis

**Affiliations:** 1 Department of Land Resources Management, China Agricultural University, Beijing, China; 2 Department of Ecology, Evolution, and Environmental Biology, Columbia University, New York, New York, United States of America; 3 The Earth Institute, Columbia University, New York, New York, United States of America; 4 The Nature Conservancy, New York, New York, United States of America; Mexican Social Security Institute, MEXICO

## Abstract

China is facing both non-communicable diseases (NCDs) and micronutrient deficiency, which have been largely related to transitions within Chinese diets, for example, the overconsumption of vegetable oils and animal-source products and decreasing consumption of coarse staple foods. In this study, we use three metrics—dietary diversity score (DDS), staple diversity score (SDS) and the proportion of coarse staple consumption (PoCS)- to investigate overall dietary transitions as well as trends in staple food consumption for nine provinces in China from 1997 to 2009. We also investigated how household characteristics, community urbanicity, and provincial conditions have affected household diets and the relationship between overall diet and staple diet across socio-economic gradients. Overall dietary diversity (DDS) showed consistent growth across all the provinces and subpopulations and was strongly associated with a household’s socio-economic status. However, staple indicators (SDS and PoCS) showed notable difference both geographically and socio-economically. The relationship between overall dietary indicator (DDS) and staple indicators (SDS, PoCS) across SES gradients revealed that education is a more important influence than income in ensuring dietary balance and nutritional quality. Our findings show that programs aimed at promoting dietary balance and healthy staple diets must account for differences between provinces in terms of agronomic, nutritional, social, and economic conditions. By identifying the socio-economic characteristics and locations of the most nutritionally vulnerable populations, this study also points toward the need for policies that incorporate nutritional considerations into grain production systems and provide a strategy for enhancing China’s national food security.

## Introduction

China, the world’s most populous country, has undergone significant transitions in terms of economic development and dietary patterns during the last several decades. Like other developing countries, the ongoing modernization of China’s food systems has had profound effects on the diets of its citizens [1]. As a result, a country that once faced widespread food insecurity and extensive undernutrition in past decades must now also contend with the emerging health burdens of obesity and other diet-related non-communicable diseases (NCDs) (e.g., type-2 diabetes, hypertension, stroke, and coronary heart disease) [2–8]. Concurrent with the proliferation of NCDs are persistent micronutrient deficiencies such as iron, vitamin A, and zinc, mainly among poor women and children. Therefore, China is facing the dual challenges of undernutrition and overnutrition at the same time, and effective responses are urgently needed to deal with the social and economic burdens posed by these food security challenges.

The emergence of many of these diet-related diseases has been attributed to dietary changes in China, which has witnessed the increasing consumption of vegetable oils, animal-source foods (pork, eggs, dairy products, etc.), and processed foods high in refined starch, sugar, salt, and unhealthy fats [2, 3, 9]. Another dietary change that has likely contributed to these challenges has been the shift of staple food consumption towards refined cereals (e.g., polished rice, white wheat) and away from traditional coarse staple foods (e.g., millet, sorghum). Staple crops (including cereals, tubers, and some legumes) provide the majority of calories in Chinese diets and serve as important sources of necessary macronutrients (e.g., carbohydrates, protein, and fiber) and key vitamins and minerals (e.g., calcium, folic acid, zinc, and iron). Because of high nutrient content of coarse staple crops relative to refined cereals, the ongoing decline in their consumption implies the decreasing nutritional quality—both in terms of richness and diversity—of staple food composition [10]. This is reflective of global trends as well, where the nutrient-to-calorie ratio in cereal consumption has declined and contributed to high levels of micronutrient deficiencies, particularly in low-income settings [10]. Given the central role of staple crops in human nutrition, agricultural production, and China’s food security at large, a thorough understanding of the trends in staple food consumption—as well as their relationship with overall dietary patterns—is essential.

In addition to these macro-scale trends, socio-economic and geographic factors can play an important role in dietary choices and food access. At the household level, diets are influenced by a variety of factors including household composition, education, occupation, and income [11, 12]. Previous research has shown that dietary quality follows a socioeconomic gradient [13], with higher-quality diets associated with greater affluence, and energy-dense, nutrient-poor diets preferentially consumed by people of lower socioeconomic status (SES) [13, 14]. Diets are also profoundly influenced by nutrition knowledge, tacit assumptions about food, and self-perception of the importance of a balanced meal [15–17]. Understanding how these various household factors influence diets can therefore help to formulate effective interventions for improving household dietary quality. Above household level, community circumstances also affect diets by influencing the availability of and access to certain foods. To account for this intermediate level of factors above the household scale, we employ a community urbanicity metric, which is a composite index that accounts for a variety of socio-economic aspects—including population, economic activity, education, infrastructure, and health and social services—that determine a community’s level of development. Lastly, in addition to these household- and community-level factors, Chinese diets vary widely across geographical settings and populations of different cultural backgrounds. This is especially true for staple food consumption, which is heavily influenced by cuisine and local production.

Against this background, we examine nine provinces with varied geographical and socioeconomic backgrounds in China to investigate: (1) the trends in overall dietary patterns and staple food consumption from 1997 to 2009 across different subpopulations and geographies; (2) the extent to which household characteristics (e.g., income, education) and community urbanicity are associated with household dietary choices, and (3) the changes in the relationship between overall dietary diversity indicators and staple dietary indicators across the SES gradient. In doing so, we seek to develop a comprehensive picture of recent trends in dietary patterns and staple food consumption across China and how these have been influenced by various socio-economic and geographic factors at national, regional, and local scales. Understanding these transitions at multiple scales will be essential for developing appropriate and targeted policy interventions that can best improve health and nutritional outcomes in China.

## Materials and methods

### Data

**Fig 1:**
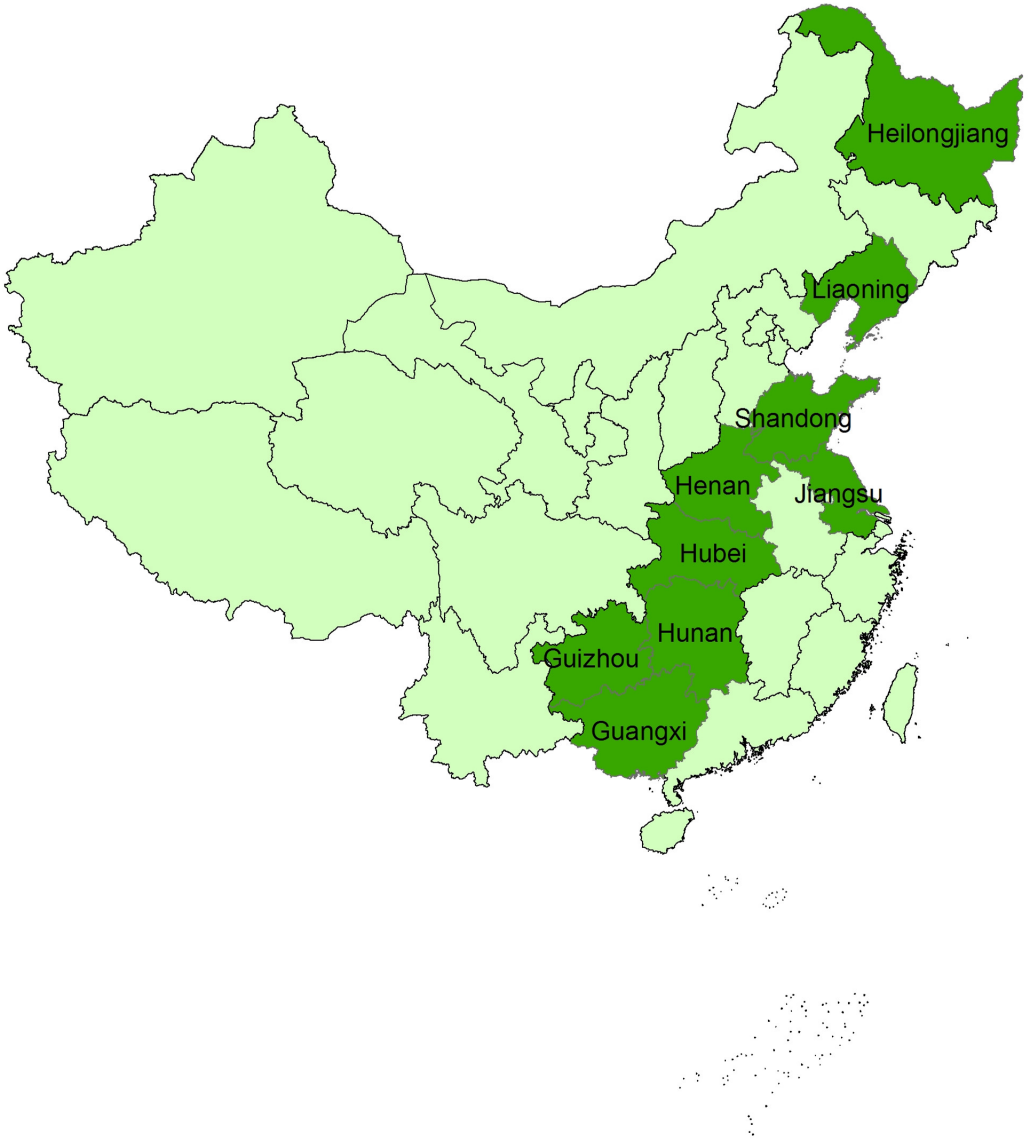
Map of the nine study provinces.

The data used in this study came from the China Health and Nutrition Survey (CHNS), a longitudinal survey designed to examine the effects of health, nutrition, and family planning policies and to see how the ongoing social and economic transformations within China have affected the health and nutritional status of its population. The survey occurred over multiple years (1997, 2000, 2004, 2006, and 2009) and covered nine provinces that vary substantially in geography, economic development, public resources, and health indicators [18]: Liaoning, Heilongjiang, Jiangsu, Shandong, Henan, Hubei, Hunan, Guangxi, and Guizhou (Fig 1). Survey sample selection was carried out using a multistage, random cluster process. Counties in each province were stratified by income (low, middle, and high), and a weighted sampling scheme was used to randomly select four counties in each province. The survey collected information on a wide array of household characteristics including demographics, economic status, time use, labor force participation, asset ownership, income, and expenditure. For the dietary intake data, the CHNS collected information based on a 3-day dietary recall, which included 2 weekdays and 1 weekend day. These data cover a total of 3490 food items, including 253 staple food items. After compiling all of these data on household dietary intake with household demographic and socioeconomic characteristics, we then performed quality checks to eliminate outliers (e.g., observations with extreme per capita income or those with a negative dietary intake value). After this data processing, the final dataset used in our study included 19381 observations from 6093 households, with some households surveyed in multiple years. Lastly, to examine the relationship between staple food production and consumption, we took data on crop- and province-specific grain production from China’s National Bureau of Statistics (NBS) [19].

### Dietary metrics

Numerous diet quality indices have been suggested to reflect different aspects of dietary quality [20] from simple tools measuring adherence to dietary recommendations to more complex indices requiring detailed analyses of macro- and micro-nutrient intakes [17]. In this study we used one dietary indicator (dietary diversity score (DDS)) and two staple food indicators (staple diversity score (SDS) and proportion of coarse staple consumption (PoCS)) to assess dietary quality at the household level.

#### Dietary diversity

Dietary diversity score (DDS) was assessed as the number of food groups consumed by each household during the survey period. This scoring method is commonly used as an effective metric of dietary quality [17, 21–28]. A diet with a high variety indicates a greater diversity in nutritional sources, and several studies have shown that the nutritional quality of a diet improves with dietary diversity [27, 29]. In this study, we categorized food items into 14 food groups: cereals; tubers and roots; legumes; vegetables; fruits; fungi; meat; eggs; fish and fish products; nuts and seeds; milk and milk products; oils and fats; sweets and sugars; and condiments and beverages. These categories cover the most commonly consumed foods in Chinese diets and reflect the diversity in nutrients provided by the different food groups. For a given household, if any member consumed any food item from one of the above mentioned categories, then that household would receive a point for that food category. Consuming different foods from the same category would not count repeatedly. Therefore, the total DDS for a household was the sum of points for the fourteen food groups, with the maximum possible score being 14.

#### Staple diversity

Staple diversity score (SDS) represents the variety of staple foods consumed by each household during the survey period. Staple foods in this study generally refer to cereals, roots, tubers, and legumes, and were categorized into 13 groups: Wheat and wheat products; rice and rice products; corn and corn products; barley; millet; sorghum; other cereals; potatoes (and products); sweet potatoes (and products); other tubers/roots; mung beans; adzuki beans; and kidney beans. For an average Chinese diet, these food groups make up more than 95% of daily per capita calories of staple food supply. Soybean were not included among the staple crops as the vast majority of soybeans are processed into tofu and other products, all of which are not staple foods in Chinese diets. When calculating a household’s SDS, a household was assigned a value of 1 if its members consumed items from at least four staple food groups; otherwise the SDS of the household was 0.

#### The proportion of coarse staple consumption

To more closely examine the nutritional quality of staple food consumption patterns, we also assessed the proportion of coarse staple consumption (PoCS) of a household, defined as the proportion of coarse staple food consumption to total staple food consumption by weight. Coarse staple foods include: corn and corn products; barley; millet; sorghum; other cereals; potato (and products); sweet potato (and products); other tubers/roots; mung beans; adzuki bean; kidney bean. Because coarse staple foods are generally rich in calories, vitamins, and minerals, their overall contribution to household staple food consumption is important to examine. A household may consume a diverse set of staple crops (as reflected in a high SDS), but if the proportion from coarse staple crops was minor relative to less nutrient-dense foods like white rice or noodles, then this could impact the nutritional quality of that household’s consumption. For all three dietary indicators, their provincial-level values were calculated as the average of the household scores for all the years of a given province.

### Staple food production

Similar to PoCS, the provincial-level proportion of coarse staple production was calculated as the ratio of the production of coarse grains (including maize, millet, sorghum and barley) in a given province to total provincial grain production. Staple production diversity was calculated using Shannon diversity index, which accounts for both the abundance and evenness of grain crops cultivated in a certain area. As shown in Eq (1), the ratio of the production of crop *i* (including paddy, wheat, maize, millet, sorghum and barley) to total grain production in the province (*pi*) was calculated, then multiplied by the natural logarithm of this proportion (ln *pi*). *R* indicated richness (i.e., the total number of crops in the area). The resulting product was summed across crops, and multiplied by -1:

$$H=-\sum_{i=1}^{R}pi\text{ln}pi$$ (1)

### Nutritional quality

To explore nutritional quality in addition to the three dietary indicators, we calculated average individual daily nutrient intake of protein, fat, fiber, iron (Fe), zinc (Zn), and calcium (Ca) for each household. Nutrient intake values were calculated by combining data on dietary records with information on nutrient content of food items in the China Food Composition Table. Nutrient Adequacy Ratio (NAR) was used to determine the adequacy of these six nutrients in diet. This information on intake was then used to quantify the nutrient adequacy ratio (NAR) of a household for each of the six nutrients, where NAR was calculated as the ratio of per capita intake of a particular nutrient to the Recommended Nutrient Intakes (RNI) from the Chinese Dietary Guidelines. We investigated the nutrient adequacy ratios (NARs) of the six nutrients in the participants’ diets by DDS, SDS and PoCS groups.

### Demographic and socio-economic variables

The multiple measures of dietary quality were examined in the context of various important household characteristics, including demographic factors (i.e., gender, age of the person preparing food, household size), as well as socioeconomic factors (i.e., average educational attainment of the family members preparing food, per capita household income). The education variable was reported as categorical variables where no education or primary school education was considered ‘low’, lower middle school and higher middle school were considered ‘medium’, and graduation from a technical school, vocational school, college, or university was considered ‘high’. Per capita income category assignments differed between rural (low: <¥2000, medium: ¥2000—¥8000, high: >¥8000) and urban (low: <¥4000, medium: ¥4000—¥13000, high: >¥13000) areas (see Table B in S1 File). We used household dependency ratio (i.e., the ratio of household members younger than 15 or older than 65 (non-labor force) to the total number of household members) to control for the effect of household composition, as households with more children and elderly might have different dietary patterns from those with more members active in the labor force [30]. At the community level, we used an urbanicity index[18] to account for the effect of community development. For a given community, this composite index assigns a score (from 1 to 10, with 10 being most developed) to twelve key components that dictate to what extent a community has fully urbanized: population density, economic activity, traditional markets, modern markets, transportation infrastructure, sanitation, communications, housing, education, diversity, health infrastructure, and social services (see Table C in S1 File)[31]. Compared to the classification of places as either urban or rural, the urbanicity index represents a more continuous and dynamic metric that can change through time and that provides a spectrum upon which a community may be placed based on characteristically urban and rural places[31]. Provincial urbanicity values were calculated as averaged community urbanicity values for all the years for each province.

### Statistical analysis

We first tested for inter-provincial differences in the means of each of the three indicators (DDS, SDS, and PoCS) by performing a series of one-way ANOVA (Analysis of Variance) tests. We also conducted pairwise comparisons to test the differences of each variable between all pairs of provinces.

We then used mixed effects logistic regression models to examine the relationship between household dietary scores and the possibility of a participant being diagnosed with hypertension, controlling for age, household per capita income, and community urbanicity index. To control for any unobserved household and county interactions, we also used household nested within county as a random effect.

Next, we fit the variables into multiple linear regression models to assess the effects of household characteristics and community urbanicity on the DDS response variable. As DDS is not a continuous variable, we utilized generalized linear mixed-effect models (GLMM) with Poisson distributions to examine the relationship of DDS with household factors and community urbanicity [32]. This regression model was first estimated for the pooled data of all provinces. In this model, in order to control for the non-independence of observations due to geographic (e.g., the same county or province) and temporal clustering (the same year), we used county nested within province and year as random effects. We assumed a different “baseline” value for each cluster by assigning different intercept values, as estimated by the mixed model. Provincial-level models were estimated with county crossed with year as the random effect. We did not include household as a random effect in the DDS models because likelihood ratio tests indicated that the models including household as a random effect were not significantly different from the ones without it.

Mixed effects logistic regression models were used to examine the association between SDS and explanatory variables. Because SDS was a binomial variable, we utilized a binomial estimator in these models. We included province as a fixed effect in the model to control the provincial effect. We included household nested within county as a random effect to account for the repeated observations of certain households and the correlation between households of the same county. The random structure was the same for each province.

For PoCS, we used a two-step procedure to estimate the effects of explanatory variables on coarse staple consumption. First, we developed a mixed effects logistic regression model to examine whether or not coarse staple crops were consumed. Second, we examined continuous PoCS variable using mixed effects models. PoCS was log-transformed to improve data skewness and kurtosis. The main reasoning for these two separate models and response variables was because determinants of the decision on whether to consume a certain kind of food often differ from the determinants of how much to consume [2, 33]. Likelihood ratios were applied to decide the best random structure of each regression model. We included both province and year as fixed effects to control the effect of province and year on PoCS, with household nested within county as the random effect.

We systematically tested for collinearity using variance inflation factors (VIFs). The VIF values for all variables were less than 1.55, well within acceptable levels [21, 34]. To facilitate comparisons across variables, all numerical predictors were standardized by subtracting their mean and dividing by their standard deviation. Response variables were left unstandardized. Partial correlations between DDS and SDS and between DDS and PoCS were performed across SES subgroups to investigate how the relationship between overall diets and staple food diets varied across a SES gradient. All statistical analyses were performed in R.

## Results

### Sample characteristics

Descriptive statistics for three response variables by year and province used in this analysis are shown in Tables 1 and 2. We observed substantial inter-provincial differences for the three dietary metrics(Table 1). For example, households in Henan province showed the lowest overall dietary diversity and relatively high SDS and PoCS. On the other hand, households in Jiangsu showed the highest DDS but relatively low PoCS.

**Table 1:** Dietary metrics by province. Mean values are shown with coefficients of variation in parentheses for DDS, SDS and PoCS. ANOVA p-values testing for differences between provinces are indicated. Asterisks (*, **, and ***) indicate statistically significant results at the 10%, 5%, and 1% level, respectively.

	*DDS*	*SDS*	*PoCS*
***Liaoning***	8.26 (0.23)	2.94 (0.36)	0.22 (0.86)
***Heilongjiang***	7.42 (0.24)	3.14 (0.28)	0.24 (0.71)
***Jiangsu***	8.77 (0.24)	2.4 (0.43)	0.10 (1.50)
***Shandong***	7.37 (0.23)	2.67 (0.39)	0.15 (1.13)
***Henan***	6.62 (0.27)	2.68 (0.41)	0.13 (1.15)
***Hubei***	6.76 (0.26)	2.08 (0.38)	0.07 (1.86)
***Hunan***	7.43 (0.20)	1.80 (0.41)	0.04 (2.5)
***Guangxi***	7.06 (0.20)	1.65 (0.41)	0.03 (2.33)
***Guizhou***	6.58 (0.23)	2.46 (0.35)	0.14 (1.43)
***P-value***	0***	0***	0 ***

**Table 2:** Dietary metrics by year. Mean values are shown with coefficients of variation in parentheses for DDS, SDS and PoCS. P-values for the slope of the trend line along years for each variable is indicated. Asterisks (*, **, and ***) indicate statistically significant results at the 10%, 5%, and 1% level, respectively.

	*DDS*	*SDS*	*PoCS*
1997	6.897 (0.258)	2.337 (0.454)	0.119 (1.487)
2000	7.046 (0.254)	2.347 (0.429)	0.119 (1.420)
2004	7.306 (0.258)	2.439 (0.407)	0.127 (1.315)
2006	7.470 (0.248)	2.421 (0.425)	0.122 (1.352)
2009	7.892 (0.239)	2.512 (0.411)	0.125 (1.296)
P-value	0.003***	0.021**	0.223

**Fig 2:**
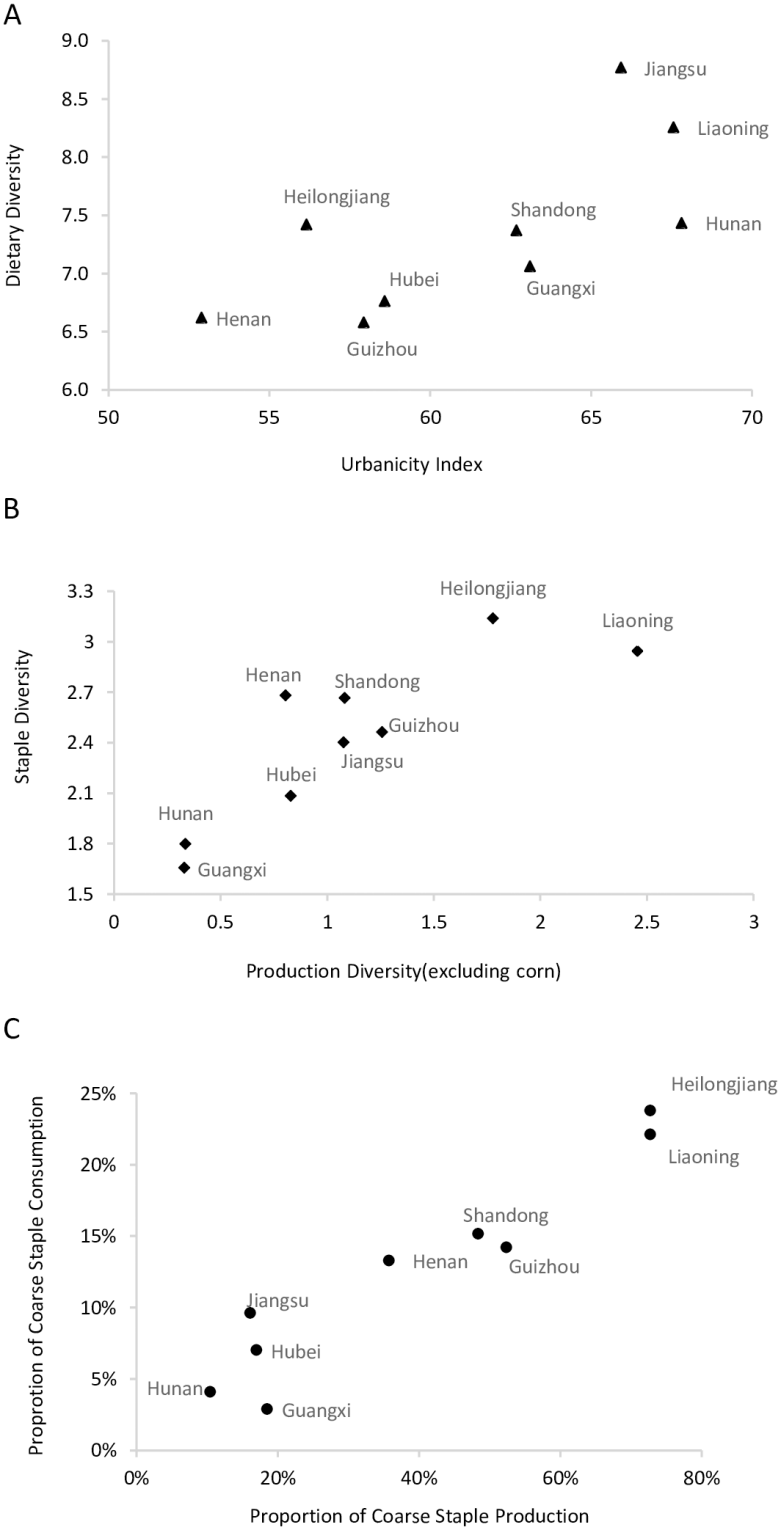
Provincial measures of dietary diversity. A) Scatter plot of dietary diversity and urbanicity index for each province); B) Staple diversity score and production diversity score for each province; and C) Proportion of coarse staple consumption and production for each province.

**Fig 3:**
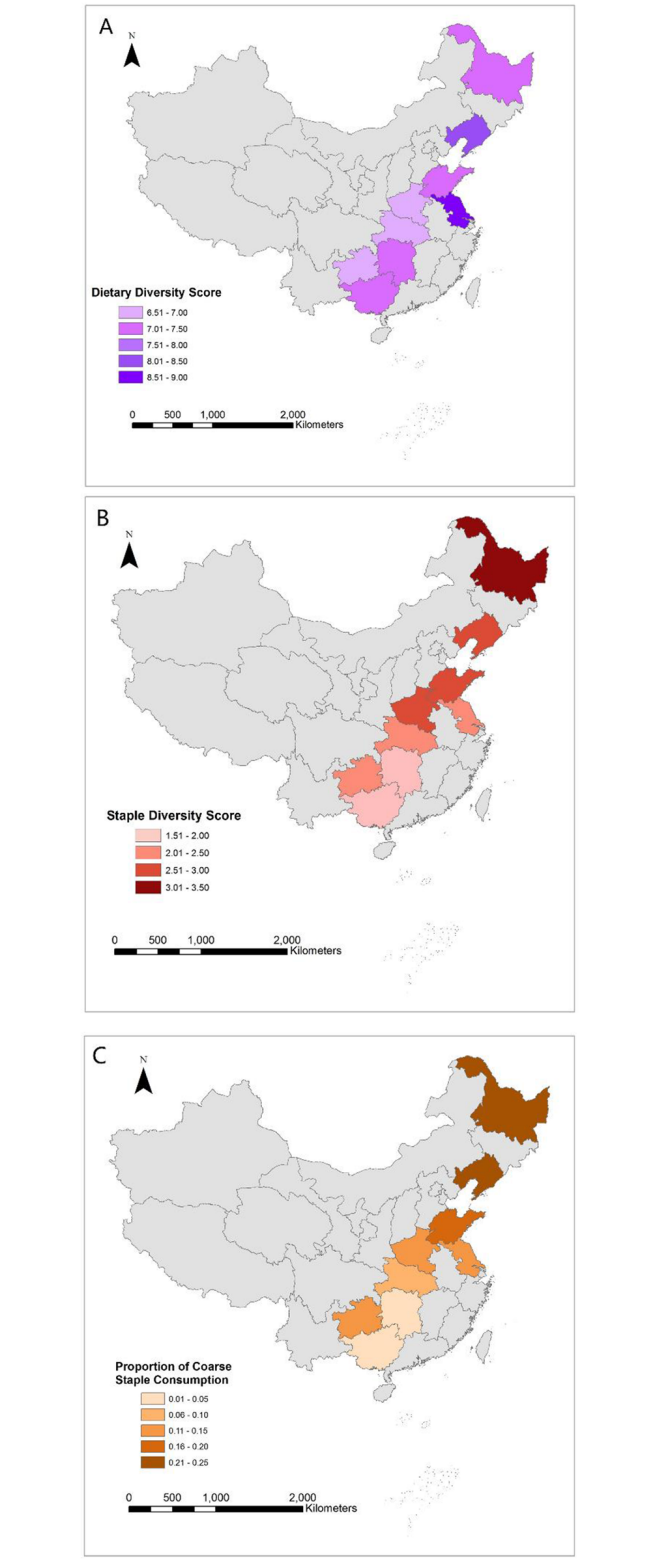
Geographic patterns of dietary diversity. Maps show A) Dietary Diversity Scores (DDS), B) Staple Diversity Scores (SDS), and C) Coarse Staple Consumption(PoCS) for the nine study provinces.

We found higher variability for PoCS and SDS relative to DDS (Tables 1 and 2), showing that there is a larger range in household staple food consumption as compared to overall patterns of dietary diversity. At the provincial level, we also found that dietary diversity is strongly correlated with urbanicity (Fig 2A). This comes as no surprise as high urbanicity generally means that a greater variety of food items are more readily available and accessible in an area. For example, Jiangsu and Liaoning show relatively high urbanicity as well as high overall dietary diversity. In contrast, Henan has the lowest urbanicty index, which is also reflected in the generally low dietary diversity scores of its households. In contrast, we found that staple indicators are more influenced by local eating habits and grain production (Fig 2B and 2C) and show significant geographical patterns (Fig 3). Liaoning and Heilongjiang in northeast China show the highest scores for staple diversity and proportion of coarse staple consumption and are also the major grain-producing areas of China. These provinces are followed by Shandong and Henan in central China, which are also dominant agricultural areas with a tradition of cultivating and consuming multiple crops. Guizhou (southwest China) also shows a relatively high staple diversity and PoCS, which mirrors its high diversity in local staple production. In southern provinces such as Hunan and Guangxi, staple production is dominated by rice, and this lack of production diversity is reflected in some of the lowest staple diversity scores among the studied provinces. Overall, all of the provinces considered in this study (except Guangxi) are major grain producers with a grain self-sufficiency ratio of more than 100% [35], meaning that each province’s grain production is sufficient to meet the dietary demands of its respective population. Therefore grain production has a direct influence on local staple consumption. Overall, northern China shows higher staple diversity and PoCS than southern China, which is largely consistent with the distribution of grain production of the country (Fig 3B and 3C). In addition to these geographic patterns, we also checked for any temporal trends, finding notable growth in dietary diversity and staple diversity (Table 2) but no significant increase for PoCS.

These three metrics provide distinct but related pictures of household diets. Taken together, they complementarily profile a more comprehensive view of dietary quality than any single metric.

Table 3 shows the NARs for protein, fat, fiber, Fe, Zn and Ca in the participants’ diets by DDS, SDS and PoCS groups. As expected, we found that the NARs of all nutrients increased with DDS. Except for fat and Zn, NARs of all nutrients improved towards 1 with increasing SDS. We found no significant difference in the NARs of macronutrients (protein and fat) across PoCS groups, while NARs for fiber, Fe, Zn, and Ca increased with PoCS. For all groups, we also observed substantial deficiencies for protein, fiber, Zn, and Ca (with NARs of all the groups well below unity). In contrast, fat intake was found to be more than the adequate intake (AI) recommended by Chinese Dietary Guidelines. Our findings also showed that these various nutritional inadequacies influence certain health outcomes. To this end, we found a significant negative association (coefficient estimate = -0.072, p = 0.0434) between PoCS and incidence of hypertension after controlling for age and community urbanicity(Table D in S1 File). This further demonstrated the importance of staple consumption in nutrition improvement.

**Table 3:** NARs of 6 individual nutrients in the participants’ diet by DDS, SDS and PoCS groups. Mean values of each group are shown with standard deviations in parentheses. Asterisks (*, **, and ***) indicate statistically significant results at the 10%, 5%, and 1% level, respectively.

	DDS				SDS				PoCS					
	Low (1–5)	Medium (6–9)	High (10–14)	P value		Low (≤3)	High (> 3)	P value		Low (0–0.33)	Medium (0.33–0.66)	High (0.66–1)	P value	
**Protein**	0.623 (0.562)	0.769 (0.965)	0.829 (0.941)	0.000 ***	+	0.743 (0.769)	0.824 (1.534)	0.000 ***	_+_	0.755 (0.790)	0.753 (1.670)	0.720 (0.583)	0.651	
**Fat**	2.054 (3.309)	2.948 (3.561)	3.256 (2.961)	0.000 ***	+	2.849 (3.259)	2.845 (4.549)	0.953		2.859 (3.165)	2.810 (5.594)	2.463 (2.161)	0.103	
**Fiber**	0.211 (0.326)	0.253( 0.316)	0.315 (0.297)	0.000***	+	0.243 (0.304)	0.326 (0.376)	0.000***	+	0.244 (0.295)	0.335 (0.432)	0.370 (0.518)	0.000 ***	+
**Fe**	1.108 (0.861)	1.213 (2.358)	1.268 (2.711)	0.006 ***	+	1.180 (2.264)	1.353 (2.123)	0.000 ***	+	1.193 (2.237)	1.271 (2.438)	1.404 (1.096)	0.040**	+
**Zn**	0.219 (0.275)	0.314 (0.469)	0.381 (0.276)	0.000 ***	+	0.308 (0.442)	0.313 (0.302)	0.563	+	0.310 (0.434)	0.307 (0.363)	0.204 (0.241)	0.005***	
**Ca**	0.399 (0.747)	0.454 (0.512)	0.544 (0.435)	0.000 ***	+	0.447 (0.484)	0.521 (0.842)	0.000 ***	+	0.454 (0.528)	0.479 (0.711)	0.515 (0.482)	0.009 ***	+

### Regression analysis on household characteristics and community urbanicity

Regression models were fit to the pooled data and used to estimate DDS, SDS, PoCS (binomial), and PoCS (%) (Table 4). We also performed regression analyses for each province (Tables E-G in S1 File).

**Table 4:** Regression results of the association between dietary metrics and household and community factors. Mean values of each group are shown with standard deviations in parentheses. Asterisks (*, **, and ***) indicate statistically significant results at the 10%, 5%, and 1% level, respectively.

	*DDS*	*SDS*	*PoCS (binomial)*	*PoCS (%)*
***Model Intercept***	1.986*** (0.03)	-0.951* (0.418)	1.134** (0.375)	0.255*** (0.016)
***Education***	0.023*** (0.0 03)	0.032 (0.031)	-0.043* (0.023)	-0.003* (0.002)
***Per Capita Income(Yuan)***	0.013*** (0.003)	0.013 (0.0249)	-0.054** (0.020)	-0.001 (0.001)
***Male***	-0.019* (0.011)	-0.295** (0.106)	-0.095 (0.073)	0.003 (0.005)
***Female***	-0.004 (0.007)	-0.101 (0.060)	-0.015 (0.046)	-0.001 (0.003)
***Age(years)***	-0.003 (0.003)	0.061* (0.030)	-0.027 (0.023)	0.003* (0.002)
***Dependency Ratio***	-0.001 (0.003)	-0.068* (0.026)	-0.051** (0.020)	0.001 (0.001)
***Household Size***	0.034*** (0.003)	0.231*** (0.026)	0.241*** (0.020)	-0.009*** (0.001)
***Urbanicity Index***	0.073*** (0.004)	0.066* (0.033)	0.009 (0.026)	-0.013*** (0.002)

We found that socioeconomic factors (i.e., education of people preparing food, per capita income of the household (after inflation to 2011)) had a positive effect on dietary diversity but a negative effect on coarse staple consumption. This implies that households with higher social-economic status consume diets that are more diverse overall but less variety and volume in coarse staple foods.

Among demographic factors, gender of the person preparing food had a significant impact on dietary and staple diversity. Compared to households with both male and female members preparing food, households with only one gender preparing food showed significantly lower dietary and staple diversity, the impacts of which were particularly pronounced for households with male-only food preparation. This suggests that encouraging both male and female members of household to participate in meal preparation might be a way to improve household staple diversity. In addition to gender, the age of the person preparing food was also positively related to staple diversity and the proportion of coarse staple consumed, showing that the participation of older household members in cooking can also contribute to the variety and proportion of coarse staple food consumed by a family. However, the effect of age on coarse staple consumption varied substantially across provinces. In Jiangsu, where multiple crops are cultivated, households with elders preparing food had a higher possibility to consume coarse staple; in Hunan, where rice is the traditionally dominant staple food, this relationship between age and coarse staple consumption had less of an effect (Table G in S1 File). Among the other demographic measures, household size had a positive effect on dietary and staple diversity, meaning larger households tend to consume more kinds of foods as well as staple foods. This might be explained by the greater quantities of food required to support large households and the variety of family members’ preferences. This relationship was less clear for coarse staple consumption, with larger households being more likely to consume coarse staple foods but less likely to have a high percentage of PoCS. At the community level, urbanicity positively contributed to dietary and staple diversity, because higher urbanized communities have enhanced availability of and access to diverse kinds of foods; However, households in highly urbanized communities also tended to consume lower proportions of coarse staple foods. To check the robustness of the models, we utilized a stepwise regression (via backward elimination) to iteratively drop insignificant variables and to identify the most parsimonious regression model. Doing so showed minimal change in the regression coefficient estimates of the reduced models (Tables I—L in S1 File), thus supporting the robustness of our results. As a further check of robustness, we used a Shannon diversity index as an alternative metric of dietary diversity and staple diversity. We repeated the regression models with these alternative measures for overall dietary diversity and staple diversity (Tables M—P in S1 File) and found that the results of these models were largely in agreement with those calculated for the main indicators.

### Dietary and staple transition by sub-population and region

**Fig 4:**
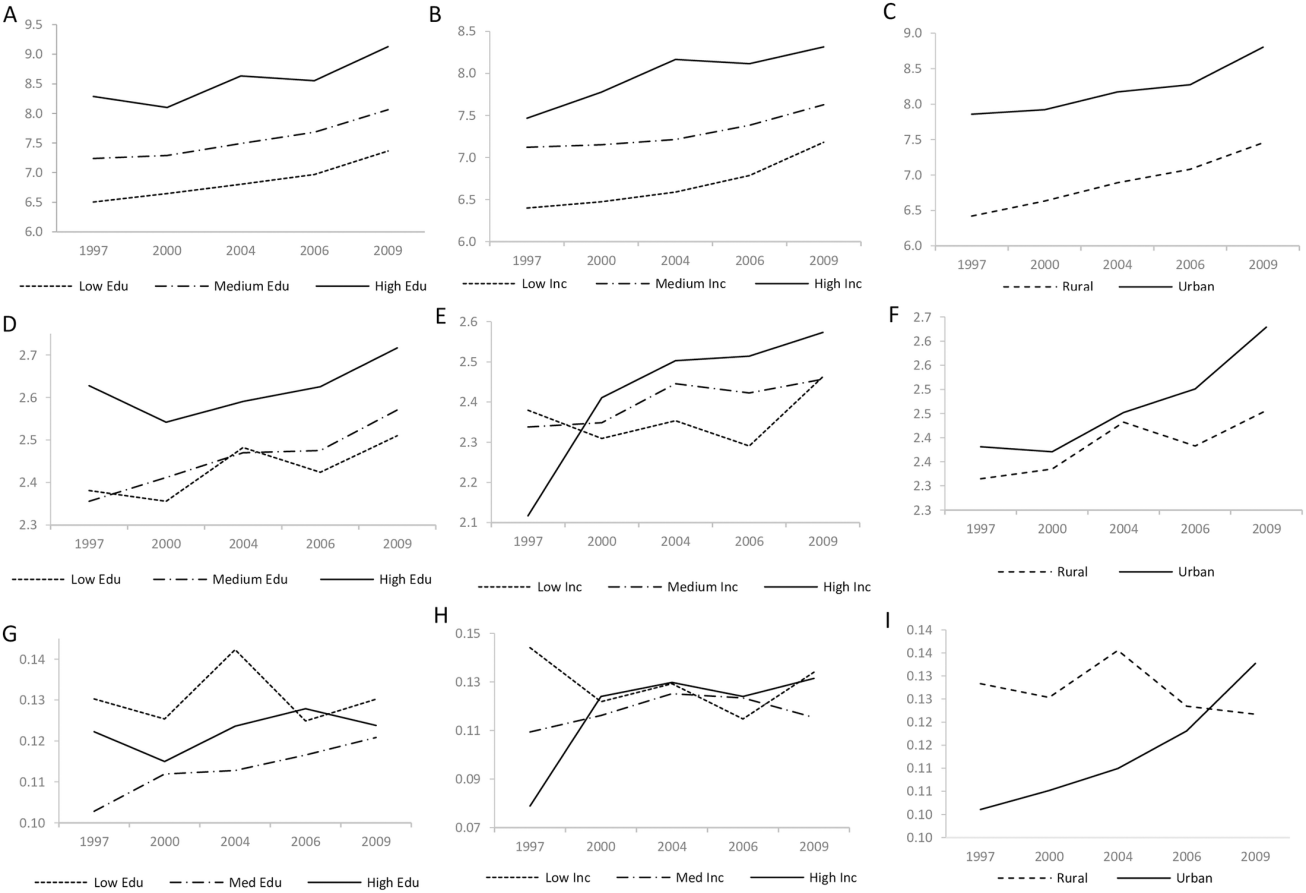
Time series of transitions in sub-group dietary diversity. Panels show changes over time by SES subgroup and rural/urban households for A/B/C) DDS, D,E,F) SDS, and G,H,I) PoCS.

Exploring in greater depth the changes across socioeconomic gradient and between rural and urban households, we found that overall dietary diversity (DDS) increased with time for all subgroups and that urban and high SES households exhibited consistently higher DDS for all study years (Fig 4A). Staple food consumption experienced a reversal across income gradient over the study period. In 1997, the higher income subgroups showed lower SDS and PoCS. The opposite was true in 2009, with higher income households showing higher SDS and PoCS (Fig 4B and 4C). A similar switch also occurred for coarse staple consumption between urban and rural households; urban households consumed a much lower PoCS in 1997 yet surpassed rural households in later years (Fig 4C).

In addition to these temporal transitions, the differences in coarse staple food consumption between rural and urban households showed significant geographical disparity (Table 1, Tables G and H in S1 File). Because of this, we decided to also explore trends in coarse staple food consumption by examining the changes in PoCS by province between rural and urban households (Table 5).

**Table 5:** Percent change in PoCS in traditionally multiple-staple-consuming provinces and rice-consuming provinces.

	Traditionally multiple-staple-consuming provinces										Rice-consuming provinces							
	Liaoning		Heilongjiang		Shandong		Henan		Guizhou		Jiangsu		Hubei		Hunan		Guangxi	
	Rural	Urban	Rural	Urban	Rural	Urban	Rural	Urban	Rural	Urban	Rural	Urban	Rural	Urban	Rural	Urban	Rural	Urban
1997	0.199	0.147	0.273	0.210	0.170	0.198	0.172	0.148	0.194	0.064	0.115	0.048	0.052	0.092	0.025	0.040	0.023	0.020
2009	0.262	0.231	0.244	0.226	0.139	0.156	0.090	0.176	0.130	0.091	0.103	0.051	0.066	0.110	0.021	0.097	0.041	0.063
Percent Change	31.48%	56.60%	-10.47%	7.62%	-18.07%	-21.50%	-47.53%	18.50%	-33.35%	41.83%	-10.21%	5.82%	28.77%	19.20%	-15.86%	138.74%	79.00%	211.22%

**Fig 5:**
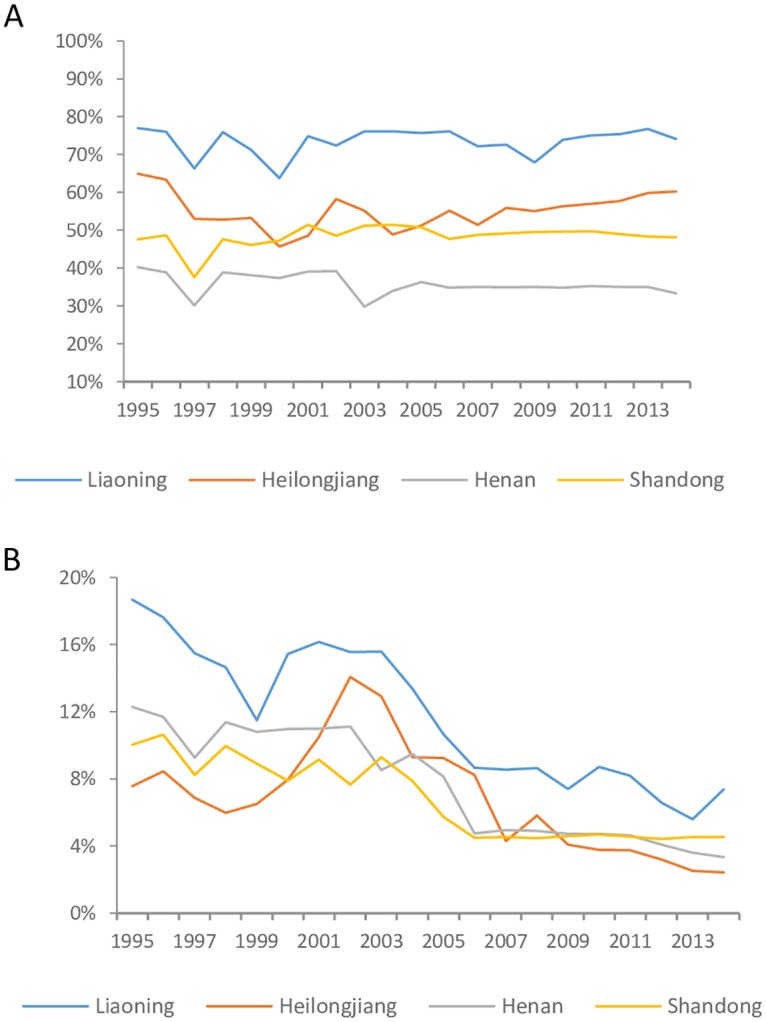
The proportion of coarse staple output in grain production before(A) and after(B) excluding corn from 1995 to 2013 in four main grain producing provinces.

We divided the nine study provinces into two groups: one with the tradition of cultivating and consuming multiple coarse staples (Liaoning, Heilongjiang, Shandong, Henan, and Guizhou), and the other with rice as the dominant staple (Jiangsu, Hunan, Guangxi and Guizhou). Table 5 shows the average rural and urban value and the relative change of PoCS (average household value) from 1997 to 2009 of each group. Rural areas in all of the provinces that have traditionally consumed multiple staple crops (except Liaoning) showed an obvious decline in coarse staple consumption. Conversely, all of the corresponding urban areas in these provinces showed an increase (except Shandong). The decrease in coarse staple food consumption in these rural areas corresponded with decreases in coarse staple production in these provinces(Fig 5). Fig 5B. shows the significant decline in the fraction of grain production contributed by coarse staple foods in four provinces (Liaoning, Heilongjiang, Henan, and Shandong) that are major producers of coarse grains. In contrast, rice-producing provinces generally showed considerable increases in coarse staple food consumption. Therefore, the homogenization of grain production in provinces that traditionally produce multiple grains has had the greatest influence on the coarse staple food consumption and staple nutritional quality of local rural households, whose rely most heavily on grains for delivering essential nutrition.

### Relationship between dietary quality and staple food quality across SES gradients

We performed partial correlations to examine the relationship between dietary diversity (DDS) and the two staple food indicators (SDS and PoCS) across education subgroups, controlling for community urbanicity, household size, and income. Partial correlation was also conducted across income subgroups and urban/rural subgroups controlling for other variables (Table 6).

**Table 6:** Correlation between dietary indicator and staple indicators. Asterisk *** indicates statistically significant results at 1% level.

		*SDS*	*PoCS*
***Dietary Diversity Score(DDS)***	Low-educated	0.236***	0.193***
	Medium-educated	0.258***	0.233***
	High-educated	0.301***	0.208***
	Low-income	0.274***	0.220***
	Medium-income	0.241***	0.207***
	High-income	0.250***	0.202***
	Urban	0.292***	0.237***
	Rural	0.201***	0.138***

Groups with higher educational attainment showed a stronger correlation between dietary diversity and staple food indicators (Table 6), implying that higher educated groups tend to have higher quality dietary compositions, with more diversified overall diets, more diversified staple food diets, and a higher proportion of coarse staple food. Thus improved educational outcomes can also promote healthy diets through improving nutritional knowledge and awareness.

Interestingly, independent of other demographic factors and education, the higher income groups showed a weaker correlation between dietary diversity and staple indicators, which means that higher-income households tend to consume more diverse overall diets but fewer staple food items and a lower proportion of coarse staple food. Urban households showed stronger positive correlations between dietary diversity and staple food indicators and therefore tend to have more diverse and balanced diets compared to rural households.

## Discussion and conclusion

### Dietary transitions in China

Our findings reveal a prevailing increase in dietary diversity and staple diversity, which can in large part be explained by overall improvements in household purchasing power and economic access to a more diverse consumption basket [36, 37]. These trends have also been influenced by a suite of interrelated factors at the aggregate level, including the rapid development of modern market systems, trade liberalization, the development of extensive commodity transport systems, multinational food industries, supermarkets, and human migration [38–42].

These overall trends in coarse staple food consumption mask interesting geographical patterns and disparities between rural and urban areas. Coarse staple food consumption declined most dramatically for rural households in areas that traditionally grow coarse grains (i.e., rural areas in Heilongjiang, Henan, Shandong, and Guizhou), dietary changes that largely reflected the substantial decreases in coarse staple food production in these areas. This transition away from coarse staple food production and consumption has been influenced by various socio-cultural and economic trends in China over the past several decades. Before economic reforms of the 1980s, most ordinary Chinese families could only afford coarse staple foods such as potato, corn, and sorghum. Eating refined cereal products like white steamed bread (Mantou) or white rice was considered a luxury. Moreover, coarse grains like corn and sorghum were widely used as animal feed, further enhancing their negative cultural perception. In part, because of these influences and perceptions, recent improvements in household purchasing power has meant that people consume substantially less coarse foods [43–45]. For rural households specifically, the decrease in coarse staple food consumption was also interrelated with the rapid decline in coarse staple production. During recent years, the cultivated extent of certain staple crops like corn and potato has rapidly expanded at the expense of minor coarse staple crops (e.g., sorghum, millet, and rye), resulting in the emerging dominance of a few major staple crops. While these relatively high-yielding crops such as corn and potato offer promise for rapidly increasing crop production, they do so at the risk of compromising the nutritional quality of diets in many households throughout China. Indeed, our findings demonstrate the importance of protecting household subsistence production of nutrient-dense grains. Decreases in the production of these minor coarse grains could adversely impact staple diversity and nutritional quality if efforts are not made to ensure that foods of equal or greater nutritional value are available for these households to purchase and consume [21, 46, 47]. Compared to the decrease in coarse staple food consumption in rural areas and in areas that traditionally grow multiple grains, we found an increase in coarse staple food consumption among urban households and households in areas that traditionally eat rice. These improvements in the diversity of staple food supply and consumption in urban areas may partly be due to the development of efficient commodity transport systems and markets. While many of the trends that we examined were to be expected, one of the most striking findings of our analysis was the reversal of the SES—coarse staple consumption relationship during the study period. In 1997, lower SES households consumed more coarse staple foods than higher SES households. These findings were consistent with previous work showing that in the 1980s and 1990s households with higher incomes were more likely to consume rice and wheat and less likely to consume other grains like millet, corn, and sorghum [8, 48]. However, our study has also demonstrated a rebound in coarse staple food consumption within high SES households and urban households, as supported by previous studies[49–51]. These findings potentially imply improving nutritional awareness among Chinese people and the potential increasing demand for coarse staple consumption in the future.

Transitions in dietary patterns and staple food consumption in China involve a complex set of influences, as entirely different outcomes can occur depending on the sub-population and location of a household. This is particularly true for the staple consumption. Only by understanding the disparity across regions and developmental trend from temporal perspective and the interconnection between them, can we have a comprehensive understanding of the transitional process and identify the the most vulnerable region and population that need most policy attention. Therefore we performed a series of analyses on geographical disparity, urban/rural difference and developmental trends of different sub-populations to break down the transitional process. By utilizing three metrics of dietary diversity in tandem, we were able to gain a more comprehensive understanding of the dietary status and dynamics of a household. For example, households in Liaoning and Jiangsu both show high overall dietary diversity, but coarse staple food consumption is much higher in Liaoning than in Jiangsu. In addition, analyses at multiple scales and across subpopulations provide distinct but related pictures of the ongoing dietary transitions across China. For example, households in higher SES groups tended to consume a lower proportion of coarse staple foods. However, the amount of coarse staple foods consumed by high SES population increased faster with time and in many cases exceeded the amounts consumed by lower SES groups.

### Influencing factors at multiple scales

This paper thoroughly investigated how household characteristics affect diets, a relationship which has been broadly studied in developed countries but for which there has been a lack of work conducted in China. At the household level, dietary diversity is strongly associated with socioeconomic factors (i.e., education and household income). Higher income means better economic access to and affordability of different kinds of food. Education is positively correlated with diet diversity, not only because education improves one’s knowledge regarding health and nutrition, but also because education lowers the cognitive cost associated with consuming variety [37, 52]. In addition, examining the dietary metrics of this study across a socio-economic gradient revealed that education plays a more important role in dietary balance and nutritional composition than income, which is supported by other studies [11]. By improving household purchasing power, dietary diversity can be improved to some extent, but dietary choices are finally determined by a household’s access to knowledge on the importance of balanced, nutritious diets [53].

Even more influential to patterns of staple food consumption are demographic factors. For example, households with both men and women preparing food showed higher diversity in SDS. Households with elders preparing food consumed more coarse staple foods in areas that traditionally cultivate multiple staple foods. Consistent with previous studies, larger households exhibited higher DDS and SDS scores but interestingly showed lower PoCS [36, 37]. Above the household level, community urbanicity had a positive effect on overall dietary diversity. For staple food consumption, however, the urbanicity effects varied across geographical areas depending on province-specific characteristics related to production, market structure, and culture. In provinces that traditionally cultivate multiple staple foods, households in less urbanized communities consumed more coarse staple foods due to the influence of local production. In rice-dominated provinces, households in more highly urbanized communities consumed more coarse staple foods, perhaps as a result of higher diversity of staple supply in local markets. Overall, local grain production played an important underlying role in dictating how community urbanicity and household characteristics affected household staple food consumption. Provincial-level analyses further illustrated this close relationship between staple consumption and grain production, which corroborates the conclusions obtained at household and community levels.

### Staple consumption and its relationship with overall diets

Our findings also build on those of existing studies by analyzing transitions in staple food consumption and dietary diversity together. This study reveals that patterns of staple food consumption have followed a different and more complicated transition pathway than overall diets. These trends reveal different patterns across subgroups and geographical areas, with some of the most substantial changes occurring within groups that most rely on staple foods for nutritional supply (i.e., rural households in areas that traditionally produce and consume multiple staple crops). Overall, Chinese diets have been shifting towards foods that are high in fat, energy dense, and nutrient poor[54]. Thus many of the dietary transitions in China seem to moving counter to those that would ensure balanced and nutritious consumption. This points to the need for greater attention on the changes in and effects of staple food composition, as these foods can greatly influence a household’s nutritional status.

### Policy recommendations

The findings of our study provide a host of insights that can help to inform policy recommendations for healthy dietary patterns and staple food consumption within China. Suitable policies should take into account the socio-economic, demographic, and geographic differences that influence a household’s dietary options and choices. Indeed, some policies and initiatives have already been undertaken to promote healthier and more balanced diets in China. The National Plan of Action for Nutrition in China approved in 1997 set out a broad range of long-term goals including the alleviation of hunger and food shortages, the improved nutritional status of the Chinese people, and the prevention and control of chronic diseases. The plan specifically proposed increasing the production of micronutrient-rich processed cereal products. In addition, the State Council published the National Program for Food and Nutrition Development (2014–2020) in 2014, which focused on effectively safeguarding food supply, optimizing food composition, and improving the nutritional status of residents. The Chinese Dietary guidelines also established principles for developing a healthy diet, including increasing the intake of whole cereals, tubers, and legumes. Our findings of improving dietary diversity in urban areas and among high SES populations suggest the success of these nutrition initiatives in recent years. As such, future dietary and nutrition interventions should seek to target low SES households which showed relatively low overall dietary diversity and little improvement in staple food consumption.

Second, we recommend gradually incorporating nutritional considerations into national food security strategies and moving beyond the objective of simply ensuring the country’s self-sufficiency in grain production. By promoting the cultivation of diverse coarse staple crops and evaluating agricultural progress based on metrics that go beyond economic cost/benefit ratios and calories per person, China can better ensure that food security and nutritional objectives are achieved [55]. With the release of the recent “National Structure Adjustment Plan for Crop Farming” released in 2016, it appears that the government has started thinking along these lines, proposing the expansion of coarse staple crops in certain areas like Northeast and Northwest China. Our study suggests that such changes would produce great benefits, especially for rural households in areas that cultivate multiple staple crops (e.g., Henan and Shandong). Aside from the nutritional benefits of crop diversification, promoting a more diverse mixture in Chinese grain production will also help to reduce system vulnerability to climate change impacts [38, 56, 57]. These enhancements of crop diversity can also have important ecological and environmental benefits.

Household diets are affected by numerous social, political, and economic factors at multiple scales, ranging from household-level decisions to community resources to national policies and international markets [55]. This study provides much needed understanding of the complex and interwoven influences of household factors, community urbanicity and regional conditions on household diets. In doing so, we have sought to identify and target the most vulnerable populations and areas that would benefit most greatly from policy interventions. Future studies could examine the changes in nutrition intake of major food items (like greens, beef, pork, milk, etc.) to gain insights into the nutritional value and health outcomes surrounding these indicators. Our work broadly shows that nutrition-related considerations are essential to incorporate into agricultural planning decisions.

## Supporting information

S1 Dataset (RAR)

S1 File (DOCX)
