# Supplementary material for: Understanding dietary and staple food transitions in China from multiple scales
Source: PLoS One. 2018 Apr 24;13(4):e0195775. doi: 10.1371/journal.pone.0195775 (PMC5915834; doi:10.1371/journal.pone.0195775)
Supplement: S1 File — (DOCX) [file pone.0195775.s002.docx]

**Table A.** Nutrient composition of staples (per 100 g)

| **Staple crops** | **Energy**  **(kcal)** | **Protein**  **(g)** | **Dietary fiber(g)** | **Vitamin A(μgRE)** | **Carotene**  **(μg)** | **Thiamin**  **(mg)** | **Riboflavin(mg)** | **Niacin**  **(mg)** | **Vitamin C(mg)** | **Vitamin E Total(mg)** | **Ca**  **(mg)** | **Mg**  **(mg)** | **Fe**  **(mg)** | **Zn**  **(mg)** | **Description**  **(item number)** |
| --- | --- | --- | --- | --- | --- | --- | --- | --- | --- | --- | --- | --- | --- | --- | --- |
| Rice | 346 | 7.4 | 0.7 | NA | NA | 0.11 | 0.05 | 1.9 | NA | 0.46 | 13 | 34 | 2.3 | 1.7 | Rice, grain, 12001 |
| Corn | 335 | 8.7 | 6.4 | 17 | 100 | 0.21 | 0.13 | 2.5 | NA | 3.89 | 14 | 96 | 2.4 | 1.7 | Corn grain, yellow, 12001 |
| Barley | 307 | 10.2 | 9.9 | NA | NA | 0.43 | 0.14 | 3.9 | NA | 1.23 | 66 | 158 | 6.4 | 4.36 | Barley grain, 14101 |
| Millet | 358 | 9 | 1.6 | 17 | 100 | 0.33 | 0.1 | 1.5 | NA | 3.63 | 41 | 107 | 5.1 | 1.87 | Foxtail millet, hulled,15101 |
| Sorghum | 351 | 10.4 | 4.3 | NA | NA | 0.29 | 0.1 | 1.6 | NA | 1.88 | 22 | 129 | 6.3 | 1.64 | Sorghum, broomcorn |
| Buckwheat, hulled | 324 | 9.3 | 6.5 | 3 | 20 | 0.28 | 0.16 | 2.2 | NA | 4.4 | 47 | 258 | 6.2 | 3.62 | Buckwheat, hulled, 19005 |
| Oat flour | 366 | 12.2 | 4.6 | 3 | 20 | 0.39 | 0.04 | 3.9 | NA | 7.96 | 27 | 146 | 13.6 | 2.21 | Oat flour, broomcorn |
| Job's tears | 357 | 12.8 | 2 | NA | NA | 0.22 | 0.15 | 2 | NA | 2.08 | 42 | 88 | 3.6 | 1.68 | Adlay,"Job's tears", 19008 |
| Potato, white | 76 | 2 | 0.7 | 5 | 30 | 0.08 | 0.04 | 1.1 | 27 | 0.34 | 8 | 23 | 0.8 | 0.37 | Potato, white, 21101 |
| Sweet potato, red flesh | 99 | 1.1 | 1.6 | 125 | 750 | 0.04 | 0.04 | 0.6 | 26 | 0.28 | 23 | 12 | 0.5 | 0.15 | Sweet potato, red flesh, 21202 |
| Mung bean | 316 | 21.6 | 6.4 | 22 | 130 | 0.25 | 0.11 | 2 | NA | 10.95 | 81 | 125 | 6.5 | 2.18 | Mung bean, broomcorn,32101 |
| Adzuki bean | 309 | 20.2 | 7.7 | 13 | 80 | 0.16 | 0.11 | 2 | NA | 14.36 | 74 | 138 | 7.4 | 2.2 | Adzuki bean, 33101 |
| Kidney bean, red | 314 | 21.4 | 8.3 | 30 | 180 | 0.18 | 0.09 | 2 | NA | 7.74 | 176 | 164 | 5.4 | 2.07 | Kidney bean, red, 34104 |
| Kidney bean, variegated | 334 | 22.5 | 3.5 | NA | NA | 0.37 | 0.28 | 2.1 | NA | 6.02 | 156 | 31 | 1.7 | 1.2 | Kidney bean, variegated,34105 |
| Kidney bean, multi colored | 306 | 22.4 | 10.5 | NA | NA | NA | NA | NA | NA | NA | 349 | 197 | 8.7 | 2.22 | Kidney bean, multi colored,34106 |

Source: China Food Composition 2002[33]

**Table B.** Description of Variables

| Variable | Description | Type | Hypothesis |
| --- | --- | --- | --- |
| Dietary Diversity Score(DDS) | The number of food groupings consumed during survey | Integer | Dependent variable |
| Staple Diversity Score (SDS) | The number of staple groupings consumed during survey | Integer | Dependent variable |
| Proportion of Coarse Staple consumption (PoCS) | % coarse staple in total staple consumption | Continuous | Dependent variable |
| Gender | Gender of the cooking members:  Both-male-and-female/Male/Female | Categorical | Demographic factor |
| Age(years) | Age of the cooking person | Continuous | Demographic factor |
| Household Size | The number of household members | Continuous | Demographic factor |
| Dependency Ratio | % household members younger than 15 or older than 65 | Continuous | Control factor |
| Education | Average education level of the cooking members  (None = 0, Grad from primary = 1, Lower middle school degree = 2, higher middle school degree = 3,  Technical or vocational degree = 4,  University or college degree and above = 5)  **Recoding-education:**  Low: 0—1 Medium: 2—3 High: 4--5 | Continuous | Socioeconomic factor |
| Per Capita Income | Per capita income of the household  **Recoding-income**  **Rural:**  Low: < 2000; Medium:2000—8000; High: ≥8000  **Urban:**  Low: <4000; Medium:4000—13000; High: ≥13000 | Continuous | Socioeconomic factor |
| Community Urbanicity Index | Community urbanicity index is a composite index composed of population density, economic activity, traditional markets, modern markets, transportation infrastructure, sanitation, communications, housing, education, diversity, health infrastructure, and social services scores of the community. | Continuous | The range of urbanicity index is from 0 to 120. Each component was allotted a maximum total of 10 points. |

**Table C.** Composition of urbanicity index

| **Population density** | Total population of the community divided by community area, from official records. |
| --- | --- |
| **Economic activity** | Typical daily wage for ordinary male workers (reported by community officials) and percentage of the population engaged in non-agricultural work. |
| **Traditional markets** | Distance to the market in three categories (within the boundaries of the community, within the city but not in this community, or not within the city/village/town) and number of days of operation for eight types of market (including food and fuel markets). |
| **Modern markets** | Number of supermarkets, cafés, Internet cafés, indoor restaurants, outdoor fixed and mobile eateries, bakeries, ice cream parlors, fast food restaurants, fruit and vegetable stands, and bars within the community boundaries. |
| **Transportation infrastructure** | Most common type of road, distance to bus stop, and distance to train stop. Distance is categorized as within the community, < = 1 kilometer from the community, or > = 1 kilometer from the community. |
| **Sanitation** | Proportion of households with treated water and prevalence of households without excreta present outside the home. |
| **Communications** | Availability (within community boundaries) of a cinema, newspaper, postal service, or telephone service; percentage of households with a computer; percentage of households with a television; and percentage of households with a cell phone |
| **Housing** | Average number of days a week that electricity is available to the community, percentage of the community with indoor tap water, percentage of the community with flush toilets, and percentage of the community that cooks with gas. |
| **Education** | Average education level among adults > 21 years old. |
| **Diversity** | Variation in community education levels and variation in community income levels. |
| **Health infrastructure** | Number and type of health facilities in or nearby (≤ 12 kilometers) the community and number of pharmacies in the community. |
| **Social services** | Provision of preschool for children under three years old and availability of (offered in community) commercial medical insurance, free medical insurance, and/or insurance for women and children |

**Table D.** Regression results for the relationship between dietary indicators and occurrence of hypertension. Asterisks (*, **, and ***) indicate statistically significant results at the 10%, 5%, and 1% level, respectively.

|  | Coefficient Estimate | Standard Error | P-value |
| --- | --- | --- | --- |
| (Intercept) | -4.063 | 0.125 | 0.000*** |
| PoCS | -0.072 | 0.031 | 0.018** |
| DDS | 0.043 | 0.030 | 0.155 |
| SDS | 0.005 | 0.032 | 0.866 |
| Age | 1.565 | 0.035 | 0.000*** |
| Per Capita Income | 0.049 | 0.022 | 0.028** |
| Urbanicity Index | 0.356 | 0.035 | 0.000*** |
| 2000 | 0.343 | 0.081 | 0.000*** |
| 2004 | 0.374 | 0.079 | 0.000*** |
| 2006 | 0.371 | 0.080 | 0.000*** |
| 2009 | 0.623 | 0.080 | 0.000*** |

**Table E.** Regression results for dietary diversity score (DDS) across the study districts. The dependent variable in all models is the household dietary diversity score. Models were determined using a Poisson estimator. Coefficient estimates are shown with robust SEs in parentheses. The pooled data model was estimated with county nested within province and crossed with year as random effects. *, **, *** Statistically significant at the 10%, 5%, and 1% level, respectively.

|  | **Pooled** | **Liaoning** | **Heilongjiang** | **Jiangsu** | **Shandong** | **Henan** | **Hubei** | **Hunan** | **Guangxi** | **Guizhou** |
| --- | --- | --- | --- | --- | --- | --- | --- | --- | --- | --- |
| **Model Intercept** | 1.986***  (0.03) | 2.083***  (0.050) | 2.020***  (0.046) | 2.125***  (0.029) | 2.003***  (0.037) | 1.952***  (0.029) | 1.928***  (0.047) | 1.988***  (0.021) | 1.936***  (0.017) | 1.898***  (0.036) |
| **Male** | -0.019*  (0.011) | -0.006  (0.034) | -0.021  (0.037) | -0.010  (0.027) | -0.058  (0.040) | -0.018  (0.039) | -0.039  (0.033) | -0.027  (0.033) | 0.002  (0.028) | -0.034  (0.034) |
| **Female** | -0.004  (0.007) | -0.005  (0.022) | -0.008  (0.020) | 0.011  (0.018) | -0.016  (0.022) | -0.042*  (0.023) | -0.009  (0.023) | -0.013  (0.021) | 0.014  (0.018) | 0.005  (0.021) |
| **Education** | 0.023***  (0.0 03) | 0.016  (0.010) | 0.010  (0.011) | 0.029**  (0.009) | 0.015  (0.011) | 0.037**  (0.012) | 0.023*  (0.011) | 0.012  (0.011) | 0.027*  (0.011) | 0.0280*  (0.011) |
| **Income**  **(per capital)** | 0.013***  (0.003) | 0.013  (0.009) | 0.005  (0.008) | 0.015*  (0.008) | 0.011*  (0.006) | 0.021*  (0.011) | 0.011  (0.008) | 0.013*  (0.007) | 0.039***  (0.011) | 0.020*  (0.011) |
| **Age** | -0.003  (0.003) | -0.012  (0.012) | -0.007  (0.011) | 0.002  (0.009) | 0.000  (0.012) | -0.005  (0.011) | -0.017  (0.011) | -0.009  (0.011) | 0.003  (0.009) | 0.003  (0.010) |
| **Dependency ratio** | -0.001  (0.003) | 0.007  (0.009) | -0.004  (0.010) | 0.010  (0.008) | -0.008  (0.009) | 0.010  (0.009) | -0.006  (0.009) | -0.006  (0.009) | 0.000  (0.009) | -0.007  (0.009) |
| **Household Size** | 0.034***  (0.003) | 0.039***  (0.011) | 0.046***  (0.013) | 0.047***  (0.007) | 0.021*  (0.010) | 0.025**  (0.009) | 0.035***  (0.010) | 0.048***  (0.009) | 0.030***  (0.007) | 0.025**  (0.009) |
| **Urbanicity Index** | 0.073***  (0.004) | 0.071***  (0.013) | 0.073***  (0.011) | 0.095***  (0.010) | 0.077***  (0.014) | 0.091***  (0.012) | 0.091***  (0.011) | 0.076***  (0.011) | 0.048***  (0.009) | 0.078***  (0.011) |

**Table F.** Regression results of the association between staple diversity score and household/community factors. The dependent variable in all models is a binomial variable for staple diversity, where a 0 means that a household consumed 3 staple foods or less and a 1 represents a household consuming more than three staple food groups. Models were estimated with a binomial estimator. Coefficient estimates are shown with robust SEs in parentheses. The pooled data model was estimated with province as fixed effects, with households nested within county and crossed with year as random effects. *, **, *** Statistically significant at the 10%, 5%, and 1% level, respectively.

|  | **Pooled** | **Liaoning** | **Heilongjiang** | **Jiangsu** | **Shandong** | **Henan** | **Hubei** | **Hunan** | **Guangxi** | **Guizhou** |
| --- | --- | --- | --- | --- | --- | --- | --- | --- | --- | --- |
| **Model intercept** | -0.951*  (0.418) | -1.022***  (0.306) | -0.993***  (0.326) | -2.342***  (0.630) | -1.313***  (0.232) | -1.536**  (0.538) | -3.822***  (0.674) | -4.609***  (0.658) | -13.744***  (2.007) | -2.877***  (0.515) |
| **Male** | -0.295**  (0.106) | -0.195  (0.239) | -0.332  (0.267) | -0.559  (0.304) | -0.582*  (0.293) | 0.212  (0.262) | -0.229  (0.432) | -0.368  (0.769) | -1.447  (1.662) | -0.399  (0.363) |
| **Female** | -0.101  (0.060) | 0.001  (0.152) | 0.065  (0.136) | -0.130  (0.181) | -0.177  (0.148) | -0.319*  (0.157) | -0.315  (0.272) | -0.091  (0.398) | 0.852  (0.842) | 0.211  (0.188) |
| **Education** | 0.032  (0.031) | 0.073  (0.074) | -0.056  (0.073) | 0.158  (0.096) | 0.025  (0.075) | -0.040  (0.080) | -0.020  (0.138) | 0.192  (0.207) | 1.426*  (0.567) | 0.209*  (0.105) |
| **Per Capital Income** | 0.013  (0.0249) | 0.121*  (0.062) | 0.002  (0.060) | -0.055  (0.084) | -0.010  (0.043) | 0.038  (0.077) | -0.102  (0.141) | -0.077  (0.187) | 0.379  (0.345) | 0.007  (0.112) |
| **Age** | 0.061*  (0.030) | 0.239**  (0.084) | 0.061  (0.070) | 0.230**  (0.084) | 0.070  (0.085) | 0.045  (0.076) | -0.100  (0.159) | 0.268  (0.232) | 0.285  (0.588) | -0.072  (0.086) |
| **Dependency Ratio** | -0.068*  (0.026) | -0.016  (0.066) | -0.056  (0.066) | -0.164*  (0.078) | -0.204**  (0.063) | -0.070  (0.067) | 0.161  (0.123) | -0.042  (0.201) | -0.889  (0.695) | -0.030  (0.085) |
| **Household Size** | 0.231***  (0.026) | 0.314***  (0.079) | 0.398***  (0.082) | 0.226***  (0.058) | 0.114  (0.067) | 0.221***  (0.061) | 0.255*  (0.134) | 0.283*  (0.140) | 0.606  (0.453) | 0.096  (0.076) |
| **Urbanicity Index** | 0.066*  (0.033) | -0.130  (0.091) | 0.014  (0.075) | -0.054  (0.088) | 0.370***  (0.088) | 0.281**  (0.089) | 0.476**  (0.151) | -0.070  (0.402) | 1.394  (0.930) | -0.212*  (0.107) |

**Table G.** Regression results of the association between presence/absence of household coarse staple food consumption and household/community factors. The dependent variable in all models is a binomial variable indicating whether or not a household consumes coarse staple foods (0 representing no and 1 representing yes) . Models were estimated with a binomial estimator. Coefficient estimates are shown with robust SEs in parentheses. The pooled data model were estimated with year and province as fixed effects, with household nested within countyas random effect. *, **, *** Statistically significant at the 10%, 5%, and 1% level, respectively.

|  | **Pooled** | **Liaoning** | **Heilongjiang** | **Jiangsu** | **Shandong** | **Henan** | **Hubei** | **Hunan** | **Guangxi** | **Guizhou** |
| --- | --- | --- | --- | --- | --- | --- | --- | --- | --- | --- |
| **Model Intercept** | 1.134**  (0.375) | 0.793***  (0.222) | 2.172***  (0.283) | -0.107  (0.584) | 1.106*  (0.516) | 1.179*  (0.508) | -0.690*  (0.277) | -1.667***  (0.310) | -2.181***  (0.422) | 0.209  (0.419) |
| **Male** | -0.095  (0.073) | 0.478*  (0.268) | -0.691*  (0.270) | 0.013  (0.195) | -0.512*  (0.249) | 0.063  (0.267) | 0.141  (0.187) | 0.061  (0.223) | -0.311  (0.208) | -0.336*  (0.195) |
| **Female** | -0.015  (0.046) | 0.034  (0.161) | -0.211  (0.174) | -0.012  (0.130) | 0.128  (0.152) | -0.115  (0.158) | 0.007  (0.129) | -0.032  (0.140) | 0.068  (0.128) | -0.099  (0.122) |
| **Education** | -0.043*  (0.023) | -0.070  (0.078) | -0.230**  (0.089) | 0.021  (0.066) | -0.036  (0.075) | -0.183*  (0.078) | 0.013  (0.063) | -0.036  (0.071) | 0.113  (0.079) | 0.057  (0.067) |
| **Per Capital Income** | -0.054**  (0.020) | -0.090  (0.067) | -0.091  (0.062) | -0.206**  (0.064) | -0.045  (0.041) | -0.008  (0.078) | -0.012  (0.045) | 0.018  (0.050) | 0.010  (0.083) | -0.025  (0.070) |
| **Age** | -0.027  (0.023) | 0.048  (0.089) | 0.008  (0.089) | 0.171*  (0.069) | 0.014  (0.085) | -0.035  (0.069) | -0.069  (0.064) | -0.222**  (0.073) | 0.029  (0.064) | -0.053  (0.058) |
| **Household Size** | 0.241***  (0.020) | 0.209*  (0.090) | 0.452***  (0.117) | 0.307***  (0.053) | 0.289***  (0.079) | 0.180**  (0.059) | 0.251***  (0.056) | 0.285***  (0.059) | 0.137*  (0.056) | 0.202***  (0.053) |
| **Urbanicity Index** | 0.009  (0.026) | -0.074  (0.095) | -0.167*  (0.095) | 0.040  (0.083) | 0.079  (0.099) | 0.156  (0.081) | 0.168**  (0.062) | 0.100  (0.114) | 0.106  (0.068) | -0.172**  (0.066) |
| **Dependency Ratio** | -0.051**  (0.020) | 0.006  (0.070) | -0.104  (0.080) | -0.121*  (0.060) | -0.057  (0.060) | -0.085  (0.059) | 0.092  (0.052) | 0.015  (0.064) | -0.148*  (0.065) | -0.083  (0.051) |
| **2000** | 0.021  (0.169) |  | -0.337*  (0.201) | 0.502**  (0.165) | 0.021  (0.169) | 0.056  (0.179) | -0.178  (0.149) | 0.341  (0.186) | 0.384*  (0.201) | -0.054  (0.152) |
| **2004** | 0.219***  (0.180) | 0.633***  (0.165) | 0.065  (0.212) | 0.224  (0.172) | 0.219  (0.180) | 0.070  (0.176) | 0.542***  (0.152) | 0.485*  (0.194) | 0.667***  (0.202) | 0.467**  (0.155) |
| **2006** | 0.114**  (0.186) | 0.772***  (0.173) | 0.578*  (0.234) | 0.120  (0.180) | 0.114  (0.186) | -0.567**  (0.176) | -0.167  (0.162) | 0.424*  (0.201) | 0.611**  (0.201) | 0.210  (0.155) |
| **2009** | 0.366***  (0.196) | 1.081***  (0.187) | 0.842***  (0.247) | 0.566**  (0.188) | *0.366**  (0.196) | -0.195  (0.180) | 0.214  (0.164) | 0.371  (0.212) | 1.387***  (0.193) | 0.112  (0.159) |

**Table H.** Regression results of the association between proportion of coarse staple consumption and household/community factors. The dependent variable in all models is the proportion of coarse staple consumption (PoCS). Coefficient estimates are shown with robust SEs in parentheses. The pooled data model was estimated with province and year as fixed effects, with households nested within community as random effects. *, **, *** Statistically significant at the 10%, 5%, and 1% level, respectively.

|  | **Pooled** | **Liaoning** | **Heilongjiang** | **Jiangsu** | **Shandong** | **Henan** | **Hubei** | **Hunan** | **Guangxi** | **Guizhou** |
| --- | --- | --- | --- | --- | --- | --- | --- | --- | --- | --- |
| **Model Intercept** | 0.255***  (0.016) | 0.233***  (0.017) | 0.230***  (0.014) | 0.157***  (0.017) | 0.212***  (0.018) | 0.186***  (0.018) | 0.176***  (0.012) | 0.137***  (0.016) | 0.144***  (0.013) | 0.193***  (0.029) |
| **Male** | 0.003  (0.005) | -0.017  (0.014) | 0.017  (0.013) | 0.013  (0.013) | -0.012  (0.017) | 0.020  (0.013) | -0.007  (0.015) | 0.002  (0.018) | 0.025*  (0.013) | -0.010  (0.015) |
| **Female** | -0.001  (0.003) | 0.001  (0.009) | 0.006  (0.007) | -0.002  (0.009) | 0.005  (0.009) | 0.000  (0.008) | -0.009  (0.010) | 0.006  (0.011) | 0.001  (0.008) | -0.012  (0.009) |
| **Education** | -0.003*  (0.002) | -0.003  (0.005) | -0.003  (0.004) | -0.007  (0.005) | -0.004  (0.005) | -0.002  (0.004) | -0.001  (0.005) | 0.008  (0.006) | 0.003  (0.005) | -0.001  (0.005) |
| **Per Capital Income** | -0.001  (0.001) | -0.002  (0.004) | -0.001  (0.003) | -0.006  (0.004) | 0.002  (0.003) | 0.003  (0.004) | -0.004  (0.004) | -0.009  (0.005) | -0.010*  (0.006) | 0.002  (0.006) |
| **Age** | 0.003*  (0.002) | 0.016**  (0.005) | 0.001  (0.004) | 0.005  (0.004) | 0.005  (0.005) | 0.001  (0.004) | 0.005  (0.005) | 0.010  (0.006) | -0.001  (0.004) | 0.000  (0.004) |
| **Household Size** | **-0.009*****  **(0.001)** | -0.004  (0.005) | -0.003  (0.004) | -0.014***  (0.003) | -0.008  (0.004) | -0.009**  (0.003) | -0.015**  (0.005) | -0.014**  (0.005) | -0.007*  (0.004) | -0.008*  (0.004) |
| **Urbanicity Index** | **-0.013*****  **(0.002)** | **-0.020*****  **(0.005)** | **-0.017*****  **(0.004)** | **-0.018*****  **(0.005)** | 0.002  (0.005) | -0.004  (0.004) | 0.004  (0.004) | 0.011  (0.008) | -0.005  (0.005) | -0.038***  (0.005) |
| **Dependency Ratio** | 0.001  (0.001) | -0.003  (0.004) | -0.002  (0.003) | -0.001  (0.004) | -0.001  (0.004) | 0.002  (0.003) | 0.004  (0.004) | 0.002  (0.005) | 0.002  (0.004) | 0.006  (0.004) |
| **2000** | -0.009*  (0.004) |  | -0.015*  (0.008) | 0.013  (0.010) | -0.031**  (0.010) | -0.013  (0.009) | 0.024*  (0.012) | 0.007  (0.016) | -0.037**  (0.013) | 0.005  (0.011) |
| **2004** | -0.013***  (0.004) | 0.004  (0.010) | -0.002  (0.008) | 0.000  (0.011) | -0.039***  (0.011) | -0.028**  (0.009) | -0.014  (0.012) | -0.017  (0.016) | -0.042**  (0.013) | 0.024  (0.011) |

**Table I. Reduced model of the relationship between dietary diversity score and household/community factors.** The dependent variable in all models is dietary diversity score (DDS). Coefficient estimates are shown with robust SEs in parentheses. The pooled data model was estimated with county nested within province and year as fixed effects, with households nested within community as random effects. *, **, *** Statistically significant at the 10%, 5%, and 1% level, respectively.

|  | ***Pooled*** | ***Liaoning*** | ***Heilongjiang*** | ***Jiangsu*** | ***Shandong*** | ***Henan*** | ***Hubei*** | ***Hunan*** | ***Guangxi*** | ***Guizhou*** |
| --- | --- | --- | --- | --- | --- | --- | --- | --- | --- | --- |
| ***Model Intercept*** | 1.986***  (0.030) | 2.080***  (0.048) | 2.022***  (0.043) | 2.131***  (0.026) | 1.989***  (0.033) | 1.952***  (0.029) | 1.917***(0.045) | 1.977***  (0.012) | 1.943***(0.013) | 1.897***(0.033) |
| ***Male*** | -0.020*  (0.011) |  |  |  |  | -0.017  (0.039) |  |  |  |  |
| ***Female*** | -0.004  (0.007) |  |  |  |  | -0.042*  (0.023) |  |  |  |  |
| ***Education*** | 0.025***  (0.003) | 0.021**  (0.009) |  | 0.026***  (0.008) | 0.020**  (0.009) | 0.037***(0.010) | 0.024**  (0.010) | 0.019**  (0.009) | 0.023**  (0.010) | 0.026***(0.009) |
| ***Per capita income*** | 0.013***  (0.003) |  |  | 0.014*  (0.008) | 0.011*  (0.006) | 0.020*  (0.011) |  | 0.013*  (0.007) | 0.039***(0.011) | 0.022**  (0.011) |
| ***age*** |  |  |  |  |  |  | -0.020*  (0.011) |  |  |  |
| ***Household Size*** | 0.035***  (0.003) | 0.041***  (0.011) | 0.052***  (0.012) | 0.046***  (0.006) | 0.024**  (0.010) | 0.026***(0.008) | 0.036***(0.010) | 0.050***(0.008) | 0.030***(0.007) | 0.026***(0.008) |
| ***Urbanicity index*** | 0.072***  (0.004) | 0.069***  (0.012) | 0.081***  (0.008) | 0.097***  (0.010) | 0.075***(0.014) | 0.091***(0.011) | 0.091***(0.011) | 0.074***(0.011) | 0.051***(0.008) | 0.078***(0.010) |

**Table J. Reduced model of the relationship between staple diversity score and household/community factors.** The dependent variable in all models is the binomial variable for coarse staple food consumption. Coefficient estimates are shown with robust SEs in parentheses. The pooled data model was estimated with province and year as fixed effects, with households nested within community as random effects. *, **, *** Statistically significant at the 10%, 5%, and 1% level, respectively.

|  | ***Pooled*** | ***Liaoning*** | ***Heilongjiang*** | ***Jiangsu*** | ***Shandong*** | ***Henan*** | ***Hubei*** | ***Hunan*** | ***Guangxi*** | ***Guizhou*** |
| --- | --- | --- | --- | --- | --- | --- | --- | --- | --- | --- |
| ***Model Intercept*** | *-0.945***  *(0.418)* | *-1.050***(0.280)* | *-0.991****  *(0.305)* | *-2.304***(0.625)* | *-1.295****  *(0.235)* | *-1.565***(0.536)* | *-4.021***(0.635)* | *-4.689***(0.583)* | *-11.95***(0.005)* | *-2.762***(0.493)* |
| ***Male*** | *-0.302***(0.106)* |  |  | ***-0.581****  ***(0.302)*** | *-0.669***  *(0.286)* | *0.213*  *(0.261)* |  |  |  |  |
| ***Female*** | ***-0.123*****  ***(0.057)*** |  |  | *-0.229*  *(0.169)* | *-0.202*  *(0.139)* | *-0.289***  *(0.147)* |  |  |  |  |
| ***Education*** |  |  |  |  |  |  |  |  | *0.976****  *(0.005)* | ***0.212*****  ***(0.087)*** |
| ***Per capita income*** |  | *0.125***  *(0.060)* |  |  |  |  |  |  |  |  |
| ***age*** | *0.050**  *(0.028)* | *0.184***  *(0.072)* |  | *0.178***  *(0.077)* |  |  |  |  |  |  |
| ***Dependency Ratio*** | *-0.071***(0.026)* |  |  | *-0.151**  *(0.078)* | *-0.189****  *(0.055)* |  |  |  |  |  |
| ***Household Size*** | *0.231****  *(0.026)* | *0.321****  *(0.078)* | *0.387****  *(0.075)* | *0.236****  *(0.057)* |  | *0.204****  *(0.058)* | *0.269***  *(0.124)* | *0.278***  *(0.138)* |  | ***0.135****  ***(0.070)*** |
| ***Urbanicity index*** | *0.085****  *(0.030)* |  |  |  | *0.386****  *(0.084)* | *0.276****  *(0.081)* | *0.451****  *(0.142)* |  | *1.291****  *(0.005)* | *-0.217***  *(0.101)* |

**Table K. Reduced model of the relationship between household coarse staple food consumption and household/community factors.** The dependent variable in all models is the binomial variable for coarse staple food consumption. Coefficient estimates are shown with robust SEs in parentheses. The pooled data model was estimated with province and year as fixed effects, with households nested within community as random effects. *, **, *** Statistically significant at the 10%, 5%, and 1% level, respectively.

|  | ***Pooled*** | ***Liaoning*** | ***Heilongjiang*** | ***Jiangsu*** | ***Shandong*** | ***Henan*** | ***Hubei*** | ***Hunan*** | ***Guangxi*** | ***Guizhou*** |
| --- | --- | --- | --- | --- | --- | --- | --- | --- | --- | --- |
| *Model Intercept* | *1.114***  *(0.373)* | *0.693****  *(0.216)* | *2.212****  *(0.280)* | *-0.129*  *(0.567)* | *1.088***  *(0.509)* | *1.086***  *(0.492)* | *-0.648***  *(0.252)* | *-1.711***(0.307)* | *-2.195***(0.411)* | *0.240*  *(0.414)* |
| *Male* |  | *0.517**  *(0.267)* | *-0.732****  *(0.267)* |  | *-0.518***  *(0.246)* |  |  |  |  | *-0.346**  *(0.195)* |
| *Female* |  | *0.104*  *(0.150)* | *-0.216*  *(0.173)* |  | *0.137*  *(0.142)* |  |  |  |  | *-0.136*  *(0.115)* |
| *Education* |  |  | *-0.237****  *(0.082)* |  |  | *-0.128***  *(0.065)* |  |  |  |  |
| *Per capita income* | *-0.061***  *(0.019)* | ***-0.107****  ***(0.064)*** |  | *-0.198***(0.063)* |  |  |  |  |  |  |
| *age* |  |  |  | *0.165***  *(0.065)* |  |  |  | *-0.198***(0.062)* |  |  |
| *Household Size* | *0.248****  *(0.019)* | *0.195***  *(0.085)* | *0.448****  *(0.110)* | *0.308****  *(0.052)* | *0.286****  *(0.073)* | *0.182****  *(0.056)* | *0.262****  *(0.052)* | *0.280****  *(0.058)* | *0.158****  *(0.052)* | *0.226****  *(0.050)* |
| *Urbanicity index* |  |  | *-0.182***  *(0.091)* |  |  | ***0.138****  ***(0.079)*** | *0.169****  *(0.058)* |  | ***0.136*****  ***(0.065)*** | *-0.157***(0.058)* |
| *Dependency Ratio* | *-0.055***  *(0.018)* |  |  | *-0.117***  *(0.060)* |  |  |  |  | *-0.170***(0.059)* | ***-0.110*****  ***(0.047)*** |
| *2000* | *0.028(0.058)* |  | *-0.343**  *(0.201)* | *0.516****  *(0.163)* | *0.032*  *(0.166)* | *0.058*  *(0.178)* | *-0.188*  *(0.148)* | *0.376***  *(0.181)* | *0.402***  *(0.200)* | *-0.054*  *(0.152)* |
| *2004* | *0.341****  *(0.058)* | *0.640****  *(0.164)* | *0.033*  *(0.209)* | *0.252*  *(0.166)* | *0.248*  *(0.171)* | *0.070*  *(0.175)* | *0.518****  *(0.150)* | *0.541****  *(0.181)* | *0.684****  *(0.201)* | *0.460****  *(0.155)* |
| *2006* | *0.167***  *(0.059)* | *0.770****  *(0.170)* | *0.539***  *(0.229)* | *0.155*  *(0.170)* | *0.145*  *(0.169)* | *-0.562***(0.174)* | *-0.201*  *(0.159)* | *0.500****  *(0.179)* | *0.646****  *(0.200)* | *0.196*  *(0.154)* |
| *2009* | *0.461****  *(0.060)* | *1.084****  *(0.183)* | *0.759****  *(0.234)* | *0.601****  *(0.176)* | *0.384***  *(0.172)* | *-0.193*  *(0.178)* | *0.172*  *(0.159)* | ***0.463*****  ***(0.181)*** | *1.400****  *(0.189)* | *0.086*  *(0.154)* |

**Table L. Reduced model of the association between the proportion of coarse staple consumption and household/community factors.** The dependent variable in all models is the proportion of coarse staple consumption (PoCS). Coefficient estimates are shown with robust SEs in parentheses. The pooled data model was estimated with province and year as fixed effects, with households nested within community as random effects. *, **, *** Statistically significant at the 10%, 5%, and 1% level, respectively.

|  | ***Pooled*** | ***Liaoning*** | ***Heilongjiang*** | ***Jiangsu*** | ***Shandong*** | ***Henan*** | ***Hubei*** | ***Hunan*** | ***Guangxi*** | ***Guizhou*** |
| --- | --- | --- | --- | --- | --- | --- | --- | --- | --- | --- |
| ***Model Intercept*** | *0.255****  *(0.016)* | *0.231****  *(0.015)* | *0.233****  *(0.011)* | *0.158****  *(0.016)* | *0.213****  *(0.016)* | *0.186****  *(0.018)* | *0.169****  *(0.009)* | *0.140****  *(0.013)* | *0.146****  *(0.013)* | *0.183****  *(0.029)* |
| ***Male*** |  |  |  |  |  | ***0.022****  ***(0.013)*** |  |  | *0.024**  *(0.013)* |  |
| ***Female*** |  |  |  |  |  | *0.003*  *(0.007)* |  |  | *-0.001*  *(0.007)* |  |
| ***Education*** | *-0.003***  *(0.001)* |  |  | ***-0.008****  ***(0.004)*** | ***-0.006****  ***(0.004)*** |  |  |  |  |  |
| ***Per Capital Income*** |  |  |  |  |  |  |  |  | *0.009**  *(0.006)* |  |
| ***Age*** | *0.004****  *(0.001)* | *0.016****  *(0.005)* |  |  |  |  | ***0.008****  ***(0.004)*** |  |  |  |
| ***Household Size*** | *-0.008***(0.001)* |  |  | ***-0.015***(0.003)*** | ***-0.008*****  ***(0.004)*** | *-0.009***(0.003)* | *-0.014****  *(0.005)* | *-0.014***(0.004)* | *--0.006**  *(0.004)* | *-0.008***  *(0.004)* |
| ***Urbanicity Index*** | *-0.013****  *(0.002)* | *-0.022***  **(0.005)* | *-0.019****  *(0.003)* | *-0.017***(0.005)* |  |  |  | ***0.013****  ***(0.007)*** |  | *-0.038***(0.005)* |
| ***Dependency Ratio*** |  |  |  |  |  |  |  |  |  | ***0.006****  ***(0.004)*** |
| ***2000*** | *-0.009***  *(0.004)* |  | *-0.013*  *(0.008)* | *0.012(0.010)* | *-0.029****  *(0.010)* | *-0.014*  *(0.009)* | *0.024***  *(0.012)* | *0.008*  *(0.016)* | *-0.037***(0.013)* | *0.006*  *(0.011)* |
| ***2004*** | *-0.013***  *(0.004)* | *0.005*  *(0.010)* | *0.00*  *(0.008)* | *-0.001*  *(0.010)* | *-0.036***(0.010)* | *-0.030***(0.009)* | *-0.014*  *(0.011)* | *-0.016*  *(0.016)* | *-0.044***(0.013)* | ***0.026*****  ***(0.011)*** |
| ***2006*** | *-0.009***  *(0.004)* | *0.024***  *(0.010)* | *0.00*  *(0.008)* | *0.004*  *(0.011)* | *-0.043***(0.010)* | *-0.038***(0.009)* | *0(0.013)* | *0.009*  *(0.016)* | *-0.036***(0.013)* | *0.016*  *(0.011)* |
| ***2009*** | *-0.012***(0.004)* | *0.028****  *(0.010)* | *-0.008*  *(0.008)* | *-0.013*  *(0.011)* | *-0.044***(0.010)* | *-0.042***(0.009)* | *0.010*  *(0.012)* | *0.018*  *(0.017)* | *-0.013*  *(0.012)* | *-0.005*  *(0.011)* |

**Table M. Regression results of Shannon Index as the alternative measure of dietary diversity (Full model).** The dependent variable in all models is the household dietary diversity score. Coefficient estimates are shown with robust SEs in parentheses. The pooled data model was estimated with county nested within province and year as fixed effects, with households nested within community as random effects. *, **, *** Statistically significant at the 10%, 5%, and 1% level, respectively.

|  | ***Pooled*** | ***Liaoning*** | ***Heilongjiang*** | ***Jiangsu*** | ***Shandong*** | ***Henan*** | ***Hubei*** | ***Hunan*** | ***Guangxi*** | ***Guizhou*** |
| --- | --- | --- | --- | --- | --- | --- | --- | --- | --- | --- |
| ***Model Intercept*** | 1.484***(0.032) | 1.629***  (0.054) | 1.539***  (0.053) | 1.590***(0.050) | 1.502***  (0.054) | 1.380***(0.039) | 1.402***(0.055) | 1.513***(0.023) | 1.443***(0.023) | 1.426***(0.040) |
| ***Male*** | -0.006  (0.007) | 0.009  (0.025) | -0.021  (0.025) | 0.003  (0.020) | -0.018  (0.027) | 0.006  (0.026) | -0.020  (0.020) | -0.009  (0.019) | 0.000  (0.015) | -0.020  (0.020) |
| ***Female*** | -0.001  (0.004) | -0.011  (0.016) | -0.001  (0.014) | 0.011  (0.013) | -0.013  (0.016) | -0.009  (0.016) | -0.009  (0.014) | -0.018  (0.013) | 0.010  (0.010) | 0.021*  (0.012) |
| ***Education*** | 0.026***(0.002) | 0.012  (0.008) | 0.012  (0.007) | 0.032***(0.007) | 0.021**  (0.008) | 0.043***(0.008) | 0.034***(0.007) | 0.016**  (0.006) | 0.027***(0.006） | 0.028***(0.007) |
| ***Per Capital Income*** | 0.017***(0.002) | 0.025***(0.007) | 0.009  (0.006) | 0.020***(0.006) | 0.012**  (0.004) | 0.031***(0.008) | 0.011*  (0.005) | 0.011**  (0.005) | 0.037***(0.007） | 0.025***(0.007) |
| ***Age*** | -0.005**(0.002) | -0.015*  (0.008) | -0.010  (0.007) | -0.010  (0.007) | 0.005  (0.008) | -0.008  (0.007） | -0.016*  (0.007) | -0.002  (0.006) | -0.003  (0.005) | 0.002  (0.006) |
| ***Dependency Ratio*** | 0.002  (0.002) | 0.001  (0.007) | 0.006  (0.006) | 0.014**  (0.006) | -0.001  (0.006) | 0.010  (0.006) | -0.002  (0.005) | -0.008  (0.005) | 0.011**  (0.005) | -0.003  (0.005) |
| ***Household Size*** | 0.010***(0.002) | 0.022**  (0.008) | 0.018**  (0.008) | 0.028***(0.005) | -0.006  (0.007) | -0.003  (0.006) | 0.007  (0.006) | 0.023***(0.005) | 0.000  (0.004) | 0.010*  (0.005) |
| ***Urbanicity Index*** | 0.089***(0.0020) | 0.080***(0.009) | 0.095  (0.008) | 0.124***(0.008) | 0.079***  (0.009) | 0.117***(0.008) | 0.112***(0.007) | 0.043***(0.008) | 0.062***(0.005） | 0.098***(0.006) |

**Table N. Regression results of Shannon Index as the alternative measure of dietary diversity (Reduced model).** The dependent variable in all models is the household dietary diversity score. Coefficient estimates are shown with robust SEs in parentheses. The pooled data model was estimated with county nested within province and year as fixed effects, with households nested within community as random effects. *, **, *** Statistically significant at the 10%, 5%, and 1% level, respectively.

|  | ***Pooled*** | ***Liaoning*** | ***Heilongjiang*** | ***Jiangsu*** | ***Shandong*** | ***Henan*** | ***Hubei*** | ***Hunan*** | ***Guangxi*** | ***Guizhou*** |
| --- | --- | --- | --- | --- | --- | --- | --- | --- | --- | --- |
| ***Model Intercept*** | 1.483***  (0.032) | 1.621***  (0.053) | 1.539***(0.052) | 1.596***  (0.048) | 1.493***  (0.053) | 1.374***  (0.037) | 1.393***  (0.054) | 1.500***  (0.021) | 1.447***  (0.022) | 1.426***(0.040) |
| ***Male*** |  |  |  |  |  |  |  |  |  | -0.020  (0.020) |
| ***Female*** |  |  |  |  |  |  |  |  |  | 0.020*  (0.012) |
| ***Education*** | 0.026***  (0.002) | 0.014**  (0.007) | 0.016**  (0.006) | 0.033***  (0.006) | 0.019***  (0.006) | 0.046***  (0.006) | 0.034***（0.006） | 0.019***  (0.006) | 0.027***  (0.005) | 0.028***(0.006) |
| ***Per Capital Income*** | 0.017***  (0.002) | 0.025***  (0.007) |  | 0.020***  (0.006) | 0.012***  (0.004) | 0.030***  (0.008) | 0.011**  (0.005) | 0.011**  (0.005) | 0.037***  (0.007) | 0.026***(0.007) |
| ***Age*** | -0.004*  (0.002) | -0.014*  (0.008) |  |  |  |  | -0.019***(0.006) |  |  |  |
| ***Dependency Ratio*** |  |  |  | 0.010*  (0.005) |  |  |  | -0.009*  (0.005） | 0.010**  (0.004) |  |
| ***Household Size*** | 0.010***  (0.002) | 0.022***  (0.008) | 0.023***(0.008) | 0.029***  (0.005) |  |  |  | 0.023***  (0.005） |  | 0.009*  (0.005) |
| ***Urbanicity Index*** | 0.089***  (0.002) | 0.080***  (0.009) | 0.093***(0.007) | 0.123***  (0.008) | 0.080***  (0.009) | 0.116***  (0.008) | 0.111***  (0.007) | 0.043***  (0.008） | 0.063***  (0.005) | 0.098***(0.006) |

**Table O. Regression results of Shannon Index as the alternative measure of staple diversity (Full model).** The dependent variable in all models is the binomial household staple diversity score. Coefficient estimates are shown with robust SEs in parentheses. The pooled data model was estimated with county nested within province and year as fixed effects, with households nested within community as random effects. *, **, *** Statistically significant at the 10%, 5%, and 1% level, respectively.

|  | **Pooled** | **Liaoning** | **Heilongjiang** | **Jiangsu** | **Shandong** | **Henan** | **Hubei** | **Hunan** | **Guangxi** | **Guizhou** |
| --- | --- | --- | --- | --- | --- | --- | --- | --- | --- | --- |
| **Model Intercept** | 0.783***  (0.065) | 0.768***  (0.055) | 0.956***  (0.038) | 0.533***  (0.118) | 0.547***  (0.052) | 0.592***  (0.068) | 0.493**  (0.070) | 0.319***  (0.058) | 0.234***  (0.032) | 0.640***  (0.073) |
| **Male** | -0.018**  (0.009) | 0.005  (0.035) | -0.101***  (0.029) | -0.020  (0.024) | -0.066*  (0.035) | 0.025  (0.032) | -0.017  (0.027) | 0.007  (0.026) | -0.005  (0.017) | -0.011  (0.027) |
| **Female** | 0.000  (0.006) | 0.027  (0.023) | -0.023  (0.016) | -0.010  (0.016) | 0.001  (0.021) | -0.011  (0.019) | -0.010  (0.019) | 0.021  (0.017) | 0.017  (0.011) | -0.002  (0.016) |
| **Education** | -0.003  (0.003) | 0.006  (0.011) | -0.024***  (0.009) | 0.007  (0.009) | -0.002  (0.010) | -0.010  (0.010) | 0.001  (0.009) | 0.011  (0.009) | 0.013*  (0.007) | -0.005  (0.009) |
| **Per Capital Income** | -0.002  (0.002) | 0.006  (0.009) | 0.000  (0.007) | -0.008  (0.008) | 0.002  (0.006) | 0.007  (0.009) | 0.001  (0.007) | -0.009  (0.006) | -0.002  (0.008) | -0.002  (0.010) |
| **Age** | 0.006**  (0.003) | 0.047***  (0.013) | 0.003  (0.008) | 0.018**  (0.009) | 0.011  (0.011) | -0.006  (0.009) | 0.005  (0.009) | 0.009  (0.009) | 0.004  (0.006) | 0.001  (0.008) |
| **Dependency Ratio** | -0.006**  (0.002) | -0.012  (0.010) | -0.003  (0.008) | -0.009  (0.007) | -0.013*  (0.008) | 0.000  (0.008) | 0.016**  (0.007) | -0.006  (0.007) | -0.012**  (0.006) | -0.014**  (0.007) |
| **Household Size** | 0.015***  (0.003) | 0.042***  (0.012) | 0.038***  (0.010) | 0.013**  (0.006) | 0.026***  (0.009) | -0.001  (0.007) | 0.015*  (0.008) | 0.003  (0.007) | -0.001  (0.005) | 0.024***  (0.007) |
| **Urbanicity Index** | 0.022***  (0.003) | 0.004  (0.014) | -0.005  (0.009) | 0.020**  (0.010) | 0.057***  (0.012) | 0.058  (0.010) | 0.030***  (0.009) | 0.036***  (0.012) | 0.064***  (0.007) | -0.028***(0.009) |

**Table P. Regression results of Shannon Index as the alternative measure of staple diversity (Reduced model).** The dependent variable in all models is the binomial household staple diversity score. Coefficient estimates are shown with robust SEs in parentheses. The pooled data model was estimated with county nested within province and year as fixed effects, with households nested within community as random effects. *, **, *** Statistically significant at the 10%, 5%, and 1% level, respectively.

|  | ***Pooled*** | ***Liaoning*** | ***Heilongjiang*** | ***Jiangsu*** | ***Shandong*** | ***Henan*** | ***Hubei*** | ***Hunan*** | ***Guangxi*** |
| --- | --- | --- | --- | --- | --- | --- | --- | --- | --- |
| ***Model Intercept*** | 0.780***  (0.065) | 0.792***  (0.052) | 0.958***  (0.039) | 0.524***  (0.116) | 0.548*** (0.051) | 0.584***  (0.067) | 0.484***  (0.069) | 0.335***  (0.056) | 0.242***  (0.032) |
| ***Male*** | -0.017*  (0.009) |  | -0.101***  (0.028) |  | -0.065*  (0.035) |  |  |  |  |
| ***Female*** | 0.002  (0.005) |  | -0.023  (0.016) |  | 0.002  (0.019) |  |  |  |  |
| ***Education*** |  |  | -0.027***  (0.006) |  |  |  |  |  |  |
| ***Age*** | 0.007***  (0.003) | 0.041***  (0.011) |  |  |  |  |  |  |  |
| ***Household Size*** | 0.015***  (0.003) | 0.039***  (0.012) | 0.038***  (0.009) | 0.012*  (0.006) | 0.024***  (0.009) |  | 0.015**  (0.007) |  |  |
| ***Urbanicity Index*** | 0.020***  (0.003) |  |  | 0.024***  (0.009) | 0.055***  (0.012) | 0.0578**  (0.010) | 0.031***  (0.008) | 0.038***  (0.012) | 0.068***  (0.006) |
| ***Dependency Ratio*** | -0.005**  (0.002) |  |  |  |  |  | 0.017**  (0.007) |  | -0.013***  (0.005) |

**Table Q. The results of pairwise comparison on DDS**

|  | **Liaoning** | **Heilongjiang** | **Jiangsu** | **Shandong** | **Henan** | **Hubei** | **Hunan** | **Guangxi** |
| --- | --- | --- | --- | --- | --- | --- | --- | --- |
| **Heilongjiang** | 0.00 | - | - | - | - | - | - | - |
| **Jiangsu** | 0.00 | 0.00 | - | - | - | - | - | - |
| **Shandong** | 0.00 | 1.00 | 0.00 | - | - | - | - | - |
| **Henan** | 0.00 | 0.00 | 0.00 | 0.00 | - | - | - | - |
| **Hubei** | 0.00 | 0.00 | 0.00 | 0.00 | 0.03 | - | - | - |
| **Hunan** | 0.00 | 1.00 | 0.00 | 0.98 | 0.00 | 0.00 | - | - |
| **Guangxi** | 0.00 | 0.00 | 0.00 | 0.00 | 0.00 | 0.00 | 0.00 | - |
| **Guizhou** | 0.00 | 0.00 | 0.00 | 0.00 | 1.00 | 0.00 | 0.00 | 0.00 |

**Table R. The results of pairwise comparison on SDS**

|  | **Liaoning** | **Heilongjiang** | **Jiangsu** | **Shandong** | **Henan** | **Hubei** | **Hunan** | **Guangxi** |
| --- | --- | --- | --- | --- | --- | --- | --- | --- |
| **Heilongjiang** | 0.00 |  | - | - | - | - | - | - |
| **Jiangsu** | 0.00 | 0.00 | - | - | - | - | - | - |
| **Shandong** | 0.00 | 0.00 | 0.00 | - | - | - | - | - |
| **Henan** | 0.00 | 0.00 | 0.00 | 0.62 | - | - | - | - |
| **Hubei** | 0.00 | 0.00 | 0.00 | 0.00 | 0.00 | - | - | - |
| **Hunan** | 0.00 | 0.00 | 0.00 | 0.00 | 0.00 | 0.00 | - | - |
| **Guangxi** | 0.00 | 0.00 | 0.00 | 0.00 | 0.00 | 0.00 | 0.00 | - |
| **Guizhou** | 0.00 | 0.00 | 0.04 | 0.00 | 0.00 | 0.00 | 0.00 | 0.00 |

**Table S. The results of pairwise comparison on PoCS**

|  | **Liaoning** | **Heilongjiang** | **Jiangsu** | **Shandong** | **Henan** | **Hubei** | **Hunan** | **Guangxi** |
| --- | --- | --- | --- | --- | --- | --- | --- | --- |
| **Heilongjiang** | 0.00 | - | - | - | - | - | - | - |
| **Jiangsu** | 0.00 | 0.00 | - | - | - | - | - | - |
| **Shandong** | 0.00 | 0.00 | 0.00 | - | - | - | - | - |
| **Henan** | 0.00 | 0.00 | 0.00 | 0.00 | - | - | - | - |
| **Hubei** | 0.00 | 0.00 | 0.00 | 0.00 | 0.00 | - | - | - |
| **Hunan** | 0.00 | 0.00 | 0.00 | 0.00 | 0.00 | 0.00 | - | - |
| **Guangxi** | 0.00 | 0.00 | 0.00 | 0.00 | 0.00 | 0.00 | 0.03 | - |
| **Guizhou** | 0.00 | 0.00 | 0.00 | 0.08 | 0.08 | 0.00 | 0.00 | 0.00 |

**Table T. The results of pairwise comparison on education**

|  | **Liaoning** | **Heilongjiang** | **Jiangsu** | **Shandong** | **Henan** | **Hubei** | **Hunan** | **Guangxi** |
| --- | --- | --- | --- | --- | --- | --- | --- | --- |
| **Heilongjiang** | 0.00 | - | - | - | - | - | - | - |
| **Jiangsu** | 0.00 | 0.00 | - | - | - | - | - | - |
| **Shandong** | 0.00 | 0.00 | 1.00 | - | - | - | - | - |
| **Henan** | 0.00 | 0.00 | 1.00 | 1.00 | - | - | - | - |
| **Hubei** | 0.00 | 0.00 | 1.00 | 1.00 | 1.00 | - | - | - |
| **Hunan** | 0.00 | 0.00 | 0.00 | 0.00 | 0.00 | 0.00 | - | - |
| **Guangxi** | 0.00 | 0.00 | 0.00 | 0.00 | 0.00 | 0.00 | 1.00 | - |
| **Guizhou** | 0.00 | 0.00 | 0.00 | 0.00 | 0.00 | 0.00 | 0.00 | 0.00 |

**Table U. The results of pairwise comparison on per capita income**

|  | **Liaoning** | **Heilongjiang** | **Jiangsu** | **Shandong** | **Henan** | **Hubei** | **Hunan** | **Guangxi** |
| --- | --- | --- | --- | --- | --- | --- | --- | --- |
| **Heilongjiang** | 0.00 | - | - | - | - | - | - | - |
| **Jiangsu** | 1.00 | 0.00 | - | - | - | - | - | - |
| **Shandong** | 0.00 | 0.64 | 0.00 | - | - | - | - | - |
| **Henan** | 0.00 | 0.00 | 0.00 | 0.00 | - | - | - | - |
| **Hubei** | 0.00 | 0.00 | 0.00 | 0.00 | 1.00 | - | - | - |
| **Hunan** | 0.00 | 1.00 | 0.00 | 0.08* | 0.00 | 0.00 | - | - |
| **Guangxi** | 0.00 | 0.00 | 0.00 | 0.00 | 1.00 | 0.32 | 0.00 | - |
| **Guizhou** | 0.00 | 0.00 | 0.00 | 0.00 | 1.00 | 0.84 | 0.00 | 1.00 |

**Table V. The results of pairwise comparison on age**

|  | **Liaoning** | **Heilongjiang** | **Jiangsu** | **Shandong** | **Henan** | **Hubei** | **Hunan** | **Guangxi** |
| --- | --- | --- | --- | --- | --- | --- | --- | --- |
| **Heilongjiang** | 0.00 | - | - | - | - | - | - | - |
| **Jiangsu** | 0.00 | 0.00 | - | - | - | - | - | - |
| **Shandong** | 0.00 | 0.00 | 1.00 | - | - | - | - | - |
| **Henan** | 1.00 | 0.00 | 0.00 | 0.00 | - | - | - | - |
| **Hubei** | 1.00 | 0.00 | 0.00 | 0.00 | 1.00 | - | - | - |
| **Hunan** | 1.00 | 0.00 | 0.00 | 0.00 | 1.00 | 1.00 | - | - |
| **Guangxi** | 1.00 | 0.00 | 0.00 | 0.00 | 1.00 | 1.00 | 1.00 | - |
| **Guizhou** | 1.00 | 0.00 | 0.00 | 0.00 | 1.00 | 1.00 | 1.00 | 1.00 |

**Table W. The results of pairwise comparison on dependency ratio**

|  | **Liaoning** | **Heilongjiang** | **Jiangsu** | **Shandong** | **Henan** | **Hubei** | **Hunan** | **Guangxi** |
| --- | --- | --- | --- | --- | --- | --- | --- | --- |
| **Heilongjiang** | 0.01 | - | - | - | - | - | - | - |
| **Jiangsu** | 0.2 | 0 | - | - | - | - | - | - |
| **Shandong** | 0.08 | 0 | 1 | - | - | - | - | - |
| **Henan** | 0 | 0 | 0.01 | 0.08 | - | - | - | - |
| **Hubei** | 0.14 | 0 | 1 | 1 | 0.03 | - | - | - |
| **Hunan** | 1 | 0 | 0.68 | 0.29 | 0 | 0.48 | - | - |
| **Guangxi** | 0 | 0 | 0 | 0 | 0.18 | 0 | 0 | - |
| **Guizhou** | 0 | 0 | 0 | 0 | 0.03 | 0 | 0 | 1 |

**Table X. The results of pairwise comparison on household size**

|  | **Liaoning** | **Heilongjiang** | **Jiangsu** | **Shandong** | **Henan** | **Hubei** | **Hunan** | **Guangxi** |
| --- | --- | --- | --- | --- | --- | --- | --- | --- |
| **Heilongjiang** | 0.44 | - | - | - | - | - | - | - |
| **Jiangsu** | 0.00 | 0.00 | - | - | - | - | - | - |
| **Shandong** | 0.00 | 0.06 | 0.00 | - | - | - | - | - |
| **Henan** | 0.00 | 0.00 | 0.44 | 0.00 | - | - | - | - |
| **Hubei** | 0.00 | 0.00 | 0.00 | 0.15 | 0.00 | - | - | - |
| **Hunan** | 0.00 | 0.00 | 0.00 | 0.44 | 0.00 | 1.00 | - | - |
| **Guangxi** | 0.00 | 0.00 | 0.00 | 0.00 | 0.00 | 0.00 | 0.00 | - |
| **Guizhou** | 0.00 | 0.00 | 1.00 | 0.00 | 0.44 | 0.00 | 0.00 | 0.00 |

**Table Y. The results of pairwise comparison on urbanicity index**

|  | **Liaoning** | **Heilongjiang** | **Jiangsu** | **Shandong** | **Henan** | **Hubei** | **Hunan** | **Guangxi** |
| --- | --- | --- | --- | --- | --- | --- | --- | --- |
| **Heilongjiang** | 0.00 | - | - | - | - | - | - | - |
| **Jiangsu** | 0.03 | 0.00 | - | - | - | - | - | - |
| **Shandong** | 0.00 | 0.00 | 0.00 | - | - | - | - | - |
| **Henan** | 0.00 | 0.00 | 0.00 | 0.00 | - | - | - | - |
| **Hubei** | 0.00 | 0.00 | 0.00 | 0.00 | 0.00 | - | - | - |
| **Hunan** | 0.93 | 0.00 | 0.01 | 0.00 | 0.00 | 0.00 | - | - |
| **Guangxi** | 0.00 | 0.00 | 0.00 | 0.93 | 0.00 | 0.00 | 0.00 | - |
| **Guizhou** | 0.00 | 0.01 | 0.00 | 0.00 | 0.00 | 0.75 | 0.00 | 0.00 |
